# Recipient Cell Factors Influence Interbacterial Competition Mediated by Two Distinct Burkholderia dolosa Contact-Dependent Growth Inhibition Systems

**DOI:** 10.1128/jb.00541-21

**Published:** 2022-08-24

**Authors:** Zaria K. Elery, A. Elizabeth Oates, Tanya Myers-Morales, Erin C. Garcia

**Affiliations:** a University of Kentucky College of Medicine, Lexington, Kentucky, USA; University of Southern California

**Keywords:** two-partner secretion system, type V secretion, CDI, CdiA, interbacterial antagonism, lipopolysaccharide, LPS

## Abstract

Contact-dependent growth inhibition (CDI) systems mediate interbacterial antagonism between Gram-negative bacteria by delivering the toxic portion of a large surface protein (termed BcpA in *Burkholderia* species) to the cytoplasm of neighboring bacteria. Translocation of the antibacterial polypeptide into recipient cells requires specific recipient outer and inner membrane proteins, but the identity of these factors outside several model organisms is unknown. To identify genes involved in CDI susceptibility in the Burkholderia cepacia complex member Burkholderia dolosa, a transposon mutagenesis selection approach was used to enrich for mutants resistant to BcpA-1 or BcpA-2. Subsequent analysis showed that candidate regulatory genes contributed modestly to recipient cell susceptibility to *B. dolosa* CDI. However, most candidate deletion mutants did not show the same phenotypes as the corresponding transposon mutants. Whole-genome resequencing revealed that these transposon mutants also contained unique mutations within a three gene locus (*wabO*, BDAG_01006, and BDAG_01005) encoding predicted lipopolysaccharide (LPS) biosynthesis enzymes. *B. dolosa wabO*, BDAG_01006, or BDAG_01005 mutants were resistant to CDI and produced LPS with altered core oligosaccharide and O-antigen. Although BcpA-1 and BcpA-2 are dissimilar and expected to utilize different outer membrane receptors, intoxication by both proteins was similarly impacted by LPS changes. Together, these findings suggest that alterations in cellular regulation may indirectly impact the efficiency of CDI-mediated competition and demonstrate that LPS is required for intoxication by two distinct *B. dolosa* BcpA proteins.

**IMPORTANCE**Contact-dependent growth inhibition (CDI) system proteins, produced by many Gram-negative bacteria, are narrow spectrum antimicrobials that inhibit the growth of closely related neighboring bacteria. Here, we use the opportunistic pathogen Burkholderia dolosa to identify genes required for intoxication by two distinct CDI system proteins. Our findings suggest that *B. dolosa* recipient cells targeted by CDI systems are only intoxicated if they produce full-length lipopolysaccharide. Understanding the mechanisms underlying antagonistic interbacterial interactions may contribute to future therapeutic development.

## INTRODUCTION

Bacteria belonging to the Burkholderia cepacia complex can be isolated from soil and water and cause infections in immunocompromised individuals such as patients with cystic fibrosis (1). Inhabiting diverse polymicrobial niches, these bacteria participate in competitive and cooperative interactions with other microorganisms. Production of quorom sensing signals, bacteriocins, and secretion systems contribute to interbacterial communication and promote survival in competitive environments.

Contact-dependent growth inhibition (CDI) systems are a subset of two-partner secretion (TPS) proteins deployed by proteobacteria to mediate interbacterial competition (2). TPS systems are characterized by a “TpsA” exoprotein that is secreted across the outer membrane by a “TpsB” partner transporter (3). In CDI systems, the toxic C terminus of the TpsA exoprotein is delivered to the cytoplasm of a neighboring bacterium upon cell-to-cell contact (2). In almost all CDI systems, toxicity is due to nuclease activity of the catalytically active C terminus. Production of a cognate immunity protein prevents autoinhibition and mediates kin versus nonkin recognition (2, 4).

Data suggest that CDI systems in the β-proteobacterial *Burkholderia* genus, encoded by *bcpAIOB* (*Burkholderia* CDI system proteins), are functionally distinct from those in γ-proteobacteria (where they are termed CdiBAI) (2, 5, 6). Genes *bcpA* and *bcpB* encode the toxic exoprotein and outer membrane transporter, respectively. The gene *bcpI* encodes an allelic specific immunity protein, and *bcpO* is predicted to encode a small lipoprotein of unknown function (5). Among closely related species, BcpB sequences and the N-terminal ~90% of BcpA are conserved. Sequence variation occurs primarily in the toxic C-terminal ~300 aa of BcpA (termed BcpA-CT) and in the BcpI protein, resulting in distinct toxins that are inactivated only by their cognate immunity proteins. B. cepacia complex species Burkholderia multivorans and Burkholderia dolosa have been shown to deploy distinct CDI systems that mediate interbacterial competition (7, 8).

Delivery of CDI system effectors to recipient cells is predicted to be a multistep process that requires specific membrane-localized recipient cell factors. Studies in the model gammaproteobacterium Escherichia coli suggest that donor cell CdiA interacts with a specific outer membrane receptor on the recipient cell surface, triggering CdiA-CT release from the donor cell (9). Further translocation of CdiA-CT into the recipient cell cytoplasm requires specific inner membrane receptors (10). Variable regions of CdiA dictate which membrane receptors facilitate toxin import (10, 11). Thus, bacterial cells are only susceptible to intoxication by a particular CdiA variant if they produce the receptors necessary for toxin translocation (12). Bacterial surface structures, such as pili and overproduced capsule, have also been observed to disrupt E. coli CDI, likely by physically blocking sufficient cell-cell contact (13, 14).

Little is known about CDI system toxin translocation in species outside gammaproteobacteria. Inner membrane proteins Bth_II0599 and GltJK have been shown to mediate import of specific Burkholderia pseudomallei and Burkholderia multivorans effectors, respectively (10, 15). Alterations to Burkholderia thailandensis lipopolysaccharide (LPS) were also shown to disrupt entry of a B. pseudomallei BcpA-CT toxin (16).

Previous studies suggested that recipient cell factors affecting CDI susceptibility vary between species and among CdiA variants. To identify genes that contribute to CDI sensitivity in *B. dolosa* recipient cells, a transposon mutagenesis selection approach was used to enrich for mutants resistant to either CDI system-1 or CDI system-2. Subsequent analyses showed that *cepR*, encoding a quorom sensing regulator, contributed modestly to interbacterial toxicity, while disruptions to *B. dolosa* LPS resulted in complete resistance to both CDI systems. Overall, these findings provide insight into the complexity of CDI and highlight the role of LPS in *Burkholderia* intoxication by distinct CDI system proteins.

## RESULTS

### Selection of *B. dolosa* mutants resistant to CDI system-1 and CDI system-2.

*B. dolosa* strain AU0158 encodes three CDI systems, two of which mediate interbacterial antagonism under native gene expression in laboratory conditions (7). Toward understanding the mechanism of *Burkholderia* CDI system effector import, we sought to identify recipient cell factors necessary for susceptibility to these two active CDI systems. A *B. dolosa* mutant lacking both CDI system-1 and CDI system-2 (Δ*bcp-1* Δ*bcp-2*) was mutagenized using a miniTn5-based transposon and serially competed against *B. dolosa* donor cells that produced either CDI system-1 (Δ*bcp-2*) or CDI system-2 (Δ*bcp-1*), as previously described (15). Transposon insertion sites identified from resistant clones found one unique insertion site, in gene BDAG_00967, from individual colonies resistant to CDI system-2 (Table S5). Surprisingly, a clone with an identical transposon insertion in BDAG_00967 was also isolated from the CDI system-1 resistant pool, along with three additional genes: BDAG_02644 (*cspD*), BDAG_02714 (*hisD*), and BDAG_03544 (*cepR*) (Table S5).

### Quorum sensing regulator CepR influences CDI efficiency.

To determine the roles of the candidate genes identified in the transposon selection, chromosomal deletions were made by allelic exchange in the parental Δ*bcp-1* Δ*bcp-2* mutant. Candidate gene *cepR* encodes a quorom sensing regulator that responds to the CepI-synthesized autoinducer *N*-octanoyl l-homoserine lactone (C_8_-HSL) (17). *B. dolosa* CepR and CepI are 94% and 93% identical to the B. cenocepacia J2315 homologs (BCAM1868 and BCAM1870), respectively. CepI and CepR have been shown to autoregulate *cepI* expression (18, 19). To determine whether *B. dolosa cepR* and *cepI* contribute to quorum sensing in a similar manner to the well-characterized B. cenocepacia homologs, we used a P*_cepI_*-*lacZ* reporter. While stationary-phase cultures of Δ*bcp-1* Δ*bcp-2* bacteria showed high levels of beta-galactosidase activity, P*_cepI_*-*lacZ* activity in Δ*bcp-1* Δ*bcp-2* Δ*cepR* mutant cells was nearly undetectable (Fig. S1). Activity was decreased ~3-fold in Δ*bcp-1* Δ*bcp-2* Δ*cepI* mutant bacteria and was restored to wild-type levels with supplementation of exogenous C8-HSL. These findings indicate that *B. dolosa cepI*, *cepR*, and C8-HSL are required for *cepI* expression and suggest that this regulatory pathway functions as predicted in *B. dolosa*.

**FIG 1:**
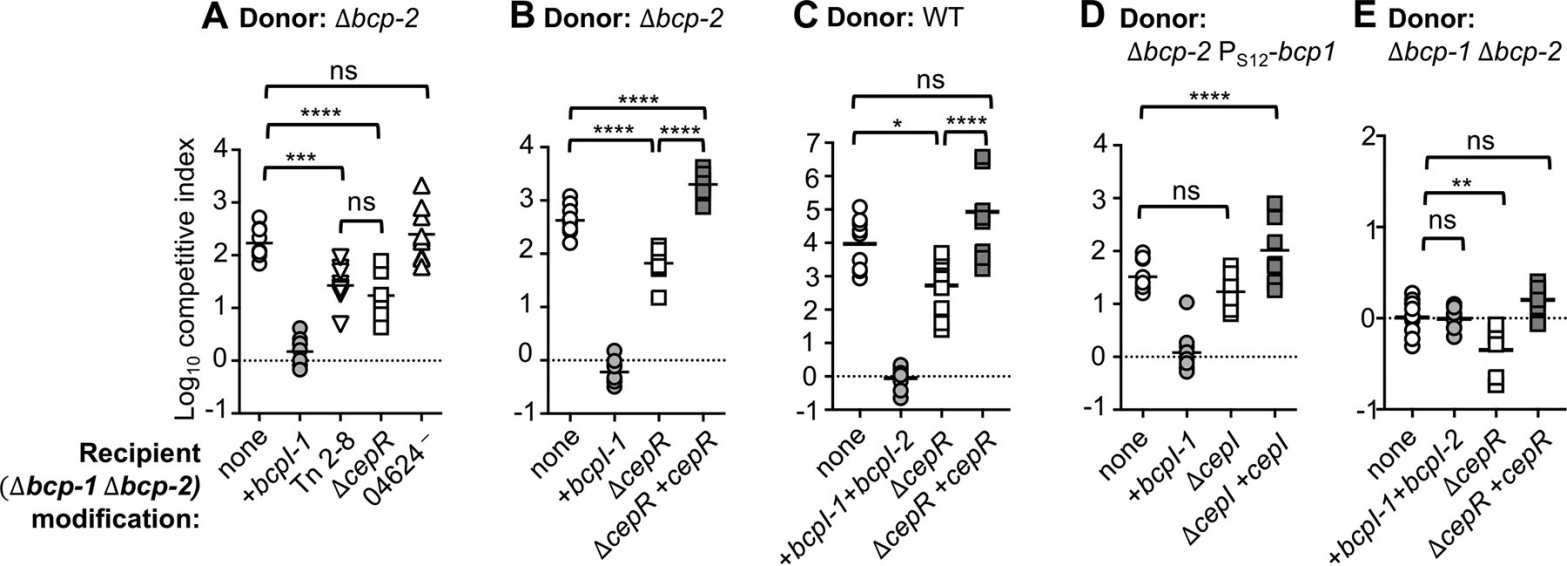
Contribution of recipient cell CepR and CepI during CDI-mediated interbacterial competition. (A, B, C, and E) Interbacterial competition assays between Δ*bcp-2* donor bacteria (A and B), wild-type (WT) donor bacteria (C), or *bcp-1 Δbcp-2* mock “donor” bacteria (E) and the indicated recipients: Δ*bcp-1* Δ*bcp-2* (parent; open circles), Δ*bcp-1* Δ*bcp-2* complemented with cognate *bcpI-1* (gray circles), *cepR*::miniTn5 transposon mutant (Tn 2-8; open inverse triangles), Δ*bcp-1* Δ*bcp-2 ΔcepR (*Δ*cepR;* open squares), Δ*bcp-1* Δ*bcp-2* containing disruption mutation in BDAG_04624 (04624^—^, open triangles), and Δ*bcp-1* Δ*bcp-2* Δ*cepR* complemented with P_S12_-*cepR* at an *att*Tn7 site (Δ*cepR* + *cepR*; gray squares). (D) Interbacterial competition assays between Δ*bcp-2* mutant overexpressing *bcpAIOB-1* (P_S12_-*bcp-1*) donor bacteria competed at a 1:10 (donor:recipient) ratio against the indicated recipients: Δ*bcp-1* Δ*bcp-2* (parent; open circles), Δ*bcp-1* Δ*bcp-2* complemented with cognate *bcpI-1* (gray circles), Δ*bcp-1* Δ*bcp-2 ΔcepI (*Δ*cepI;* open squares), or Δ*bcp-1* Δ*bcp-2* Δ*cepI* complemented with P_S12_-*cepI* at an *att*Tn7 site (Δ*cepI* + *cepI*; light gray squares). Symbols represent log_10_ competitive index values (ratio of donor to recipient) from three independent experiments and bars show the mean (*n *= 9). Dashed line shows log_10_ competitive index = 0. ns, not significant; *, *P < *0.05; **, *P < *0.01; ***, *P < *0.001; and ****, *P < *0.0001.

Quorum sensing has been shown to alter the expression of B. thailandensis *bcpAIOB* in donor bacteria (20), but the role of this regulatory network in recipient cells is unknown. Recipient bacteria (Δ*bcp-1* Δ*bcp-2*) carrying a transposon insertion or deletion in *cepR* were outcompeted by donor bacteria producing CDI system-1 (Δ*bcp-2*) to a lesser extent than the parent Δ*bcp-1* Δ*bcp-2* recipient strain (Fig. 1A). Complementation of the Δ*cepR* mutant with a constitutive copy of *cepR* restored CDI system-1 susceptibility to parent levels (Fig. 1B). Although loss of *cepR* did not impact sensitivity to CDI system-2 (Fig. S2), Δ*bcp-1* Δ*bcp-2* Δ*cepR* recipient cells were partially protected against competition from wild-type bacteria (Fig. 1C).

To further test the role of CepIR-mediated quorum sensing, we competed Δ*cepI* recipient cells against donor bacteria that overexpressed the genes encoding CDI system-1 or -2. To minimize possible complementation of Δ*cepI* recipients with autoinducer produced by the cocultured CepI+ donor cells, the competition assays were inoculated at a 1:10 (donor:recipient) ratio, as in previous studies (20). Surprisingly, no differences in competitive indices were observed for Δ*cepI* recipient cells compared to the parent strain (Fig. 1D, Fig. S2), indicating that *cepI* is not required in recipient cells. It is not clear why the Δ*cepR* and Δ*cepI* mutants displayed different CDI sensitivities, although the presence of other potentially overlapping quorom sensing networks has not been explored in *B. dolosa*.

B. thailandensis quorum sensing mutants have been shown to exhibit growth advantages in liquid medium, but not on solid medium (21). Since growth rate could impact interbacterial competition, we tested the Δ*cepR* mutant in mock competitions against the parent strain to assess its relative fitness on solid medium in the absence of CDI. When competed against Δ*bcp-1* Δ*bcp-2* bacteria, the Δ*bcp-1* Δ*bcp-2* Δ*cepR* mutant showed a small, but statistically significant competitive advantage during culture on agar (Fig. 1E), suggesting that growth rate or production of other antibacterial factors by the Δ*cepR* mutant also contributed to its enhanced survival during interbacterial competition. Although the defect of the Δ*cepR* mutant during competition against wild-type bacteria (>10-fold decrease in mean competitive index compared to the parent strain) was greater than against “mock” competitors (~3-fold decrease in competitive index compared to the parent strain), we cannot rule out the possibility that a CDI-independent mechanism is responsible for the improved survival of the Δ*cepR* mutant during interbacterial competition. Together, these results suggest that loss of the quorum sensing receptor CepR may impact *B. dolosa* competitive fitness during CDI via multiple mechanisms.

### Minimal contribution of candidate genes *cspD*, *hisD*, and BDAG_00967 to CDI susceptibility.

**FIG 2:**
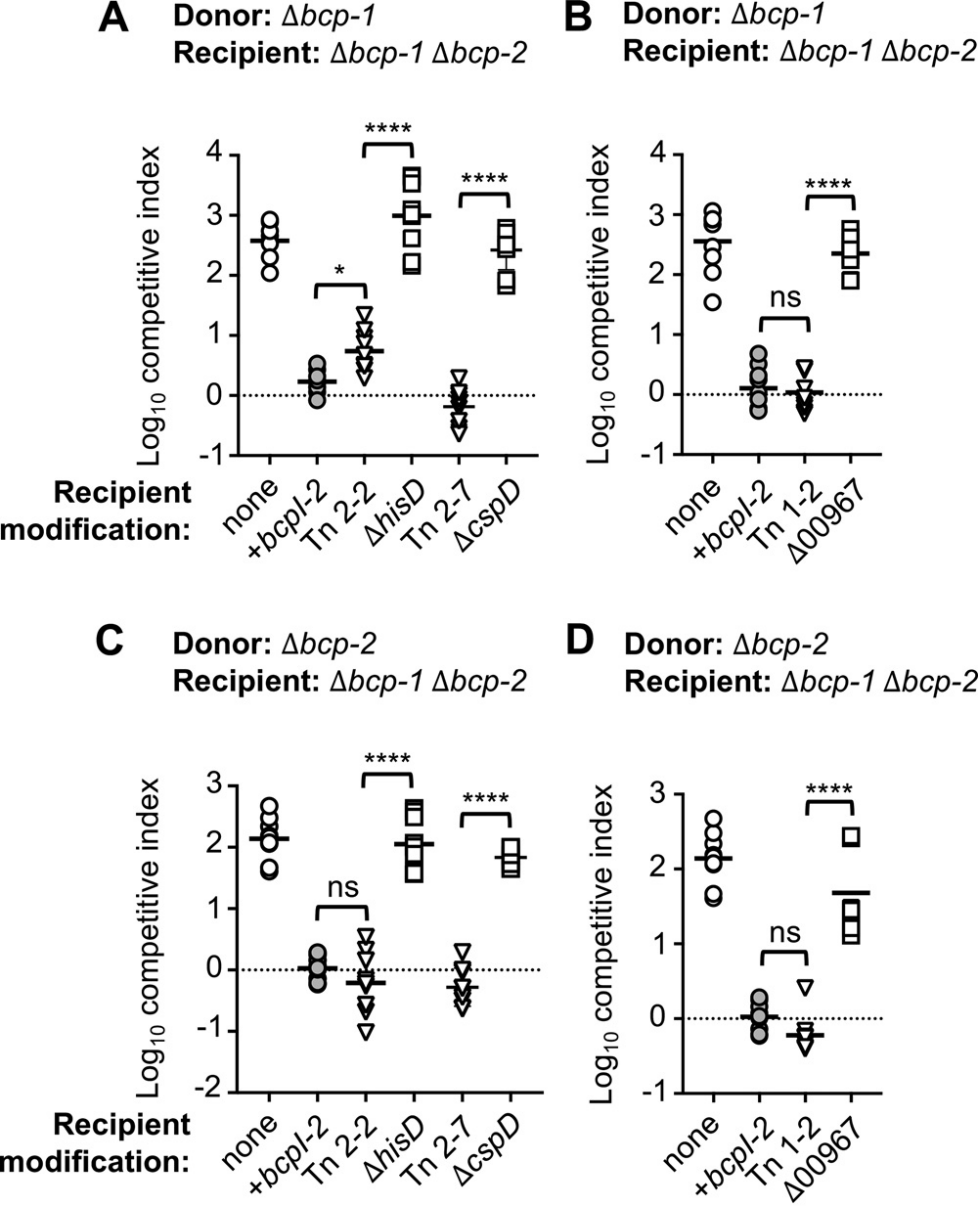
Sensitivity of *B. dolosa* transposon mutants and corresponding deletion mutants to CDI system-1 and -2. Interbacterial competition assays between Δ*bcp-1* donor bacteria (A and B) or Δ*bcp-2* donor bacteria (C and D) and the indicated control recipients: Δ*bcp-1* Δ*bcp-2* (parent; open circles) and Δ*bcp-1* Δ*bcp-2* complemented with cognate *bcpI* (light gray circles). (A and C) Recipient bacteria also include: *hisD*::miniTn5 transposon mutant, (Tn 2-2; inverse triangles) Δ*bcp-1* Δ*bcp-2* Δ*hisD (*Δ*hisD;* open squares), *cspD*::miniTn5 transposon mutant (Tn 2-7; inverse triangles), and *Δbcp-1 Δbcp-2 ΔcspD (*Δ*cspD*; open squares). (B and D) Recipient bacteria also include BDAG_00967::miniTn5 transposon mutant, (Tn 1-2; inverse triangles) and *Δbcp-1 Δbcp-2 Δ*BDAG_00967 (Δ00967; open squares). Symbols represent log_10_ competitive index values (ratio of donor to recipient) from three independent experiments and bars show the mean (*n *= 9); ns, not significant; *, *P < *0.05; and ****, *P < *0.0001.

Candidate genes *hisD* and *cspD* encode histidinol dehydrogenase and a putative cold shock-like protein, respectively. *hisD* mutants behaved as expected with respect to histidine biosynthesis (22), as both the *hisD*::miniTn5 and Δ*hisD* mutants were unable to grow in minimal medium unless supplemented with exogenous histidine (Fig. S3). However, while transposon mutants containing insertions in *hisD* and *cspD* were resistant to intoxication by both CDI system-1 and -2, the corresponding deletion mutants remained sensitive to growth inhibition (Fig. 2A and C). These results suggest that other genetic factors are responsible for the CDI resistance observed in the isolated transposon mutants.

**FIG 3:**
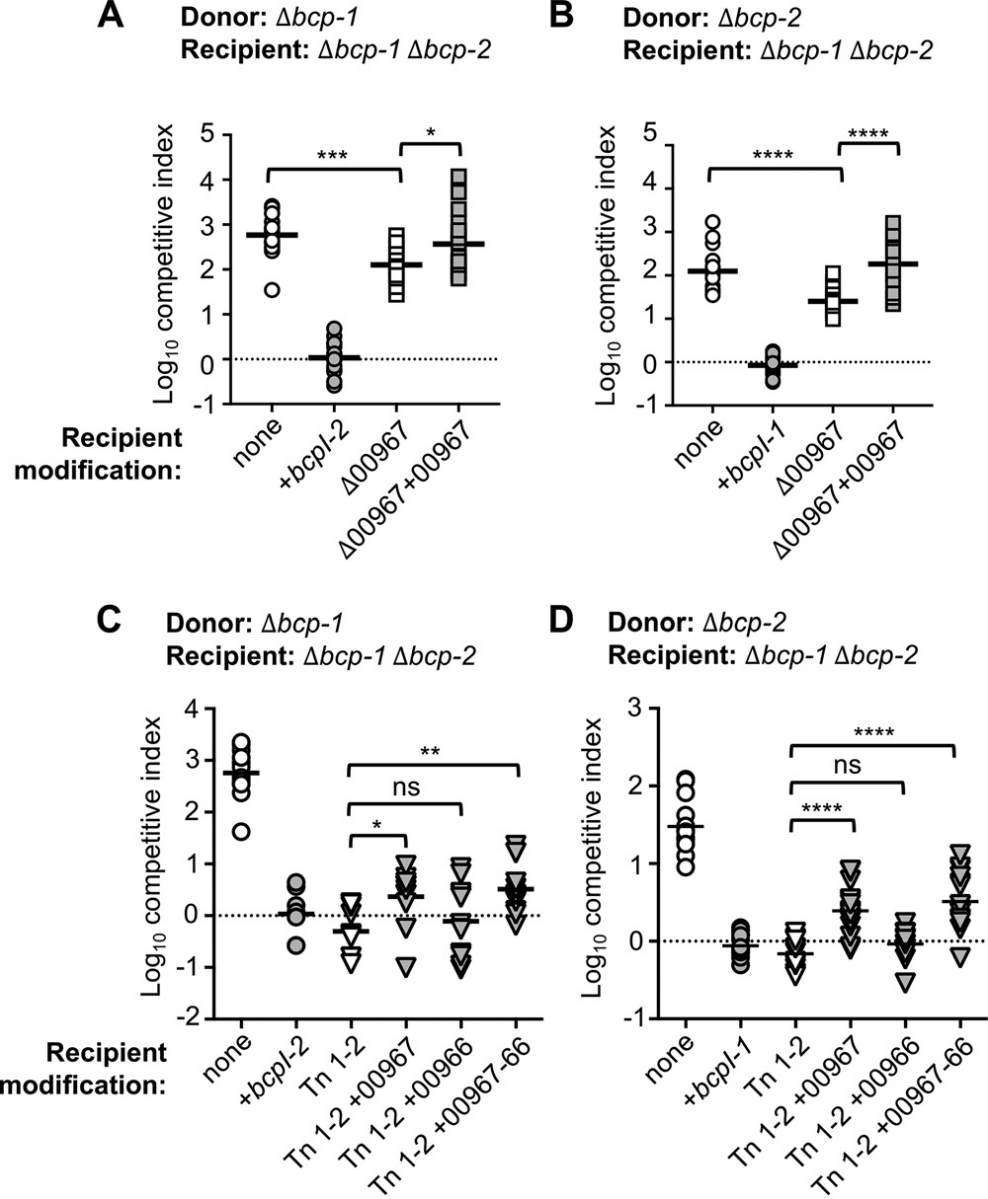
Contribution of recipient cell BDAG_00967 during CDI-mediated interbacterial competition. Interbacterial competition assays between Δ*bcp-1* (A and C) or Δ*bcp-2* (B and D) donor bacteria and the indicated recipients: Δ*bcp-1* Δ*bcp-2* (parent; open circles) and Δ*bcp-1* Δ*bcp-2* complemented with cognate *bcpI* (gray circles). (A and B) Additional recipient cells were *Δbcp-1 Δbcp-2* ΔBDAG_00967 (Δ00967; open squares) and *Δbcp-1 Δbcp-2* ΔBDAG_00967 complemented with P_S12_-BDAG_00967 at an *att*Tn7 site (Δ00967 + 00967; gray squares). Symbols represent log_10_ competitive index (ratio of donor to recipient) from six independent experiments (including replotted data, *n *= 6, from Fig. 2B and D) and bars show the mean (*n* = 17 to 18). (C and D) Additional recipient cells were BDAG_00967::miniTn5 transposon mutant, (Tn 1-2; open inverse triangles), BDAG_00967::miniTn5 transposon mutant complemented with P_S12_-BDAG_00967, P_S12_-BDAG_00967-00966 or BDAG_00966 at an *att*Tn7 site (Tn1-2 + 00967, Tn1-2 + 00966, Tn1-2 + 00967-66; gray inverse triangles). Symbols represent log_10_ competitive index (ratio of donor to recipient) from 4 to 5 independent experiments and bars show the mean (*n *=* *9 to 15). Dashed line shows log_10_ competitive index = 0; ns, not significant; *, *P < *0.05; **, *P < *0.01; ***, *P < *0.001; and ****, *P < *0.0001.

Similar results were observed for candidate gene BDAG_00967, which encodes a putative cytoplasmic membrane protein that contains GGDEF and EAL domains and is predicted to metabolize the second messenger cyclic di-GMP (c-di-GMP). While the BDAG_00967::miniTn5 transposon mutant was resistant to both CDI systems 1 and 2, ΔBDAG_00967 mutants remained sensitive to CDI (Fig. 2B and D). An approximately 25-fold reduction in mean competitive index (1.4-fold difference in log_10_ competitive index) was observed for ΔBDAG_00967 mutant recipient cells competed against donor bacteria producing either CDI system-1 or -2 (Fig. 3A and B). This phenotype was reversed by complementation with a constitutive copy of BDAG_00967, suggesting that BDAG_00967 may contribute slightly to CDI susceptibility.

To examine the possibility of transposon polarity effects, we attempted to complement the BDAG_00967::miniTn5 transposon mutant with wild-type copies of BDAG_00967 and the gene located 196 bp downstream, BDAG_00966 (encoding a putative chromate transporter). Complementation with BDAG_00967 or a construct containing both BDAG_00967 and BDAG_00966 (but not BDAG_00966 alone) had statistically significant impacts on sensitivity to CDI system-1 (Fig. 3D) and -2 (Fig. 3C). However, the CDI sensitivity of these complemented mutants was not restored to the same level as the parent strain. Together, these data indicate that BDAG_00967 expression may contribute slightly to recipient cell sensitivity to *B. dolosa* CDI, suggesting that alterations to c-di-GMP homeostasis could affect this process. However, the results also indicate that the BDAG_00967::miniTn5 transposon mutant likely contains additional mutations that are responsible for its complete CDI resistance.

### Whole-genome resequencing identifies additional unique mutations in CDI-resistant transposon mutants.

**FIG 4:**
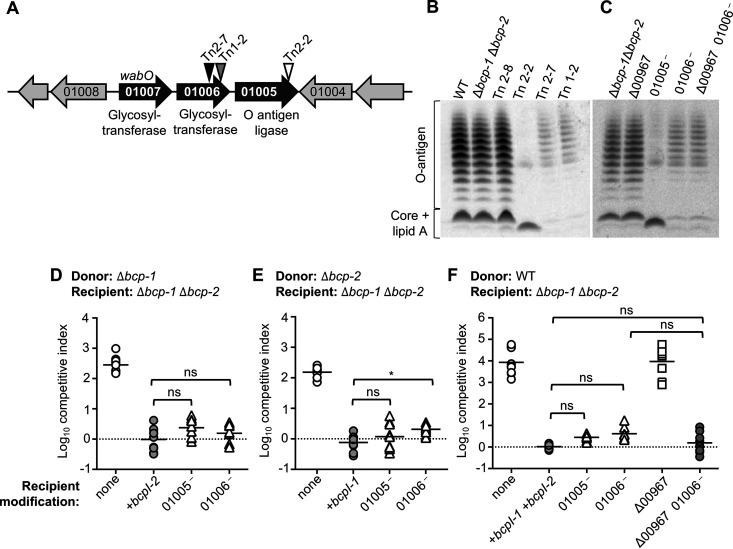
Contribution of *B. dolosa* recipient cell LPS and genes encoding putative LPS biosynthetic enzymes to susceptibility to CDI systems-1 and -2. (A) Locus organization of *wabO* (BDAG_01007), BDAG_01006, and BDAG_01005 (annotations below). Triangles denote approximate locations of additional mutations identified in transposon mutants Tn1-2 (gray), Tn2-2 (white), and Tn2-7 (black). (B) Extracted LPS separated by SDS-PAGE and stained with Pro-Q Emerald 300 from wild-type *B. dolosa* (WT), Δ*bcp-1* Δ*bcp-2* mutant (parent), and transposon mutants Tn2-8 (*cepR*::miniTn5), Tn2-2 (*hisD*::miniTn5), Tn2-7 (*cspD*::miniTn5), and Tn1-2 (BDAG_00967::miniTn5). (C) Extracted LPS separated by SDS-PAGE and stained with Pro-Q Emerald 300 from Δ*bcp-1* Δ*bcp-2* mutant, *Δbcp-1 Δbcp-2 Δ*BDAG_00967 (*Δ*00967), *Δbcp-1 Δbcp-2* containing disruption mutations in BDAG_01005 (01005^—^) or BDAG_01006 (01006^—^), and Δ*bcp-1 Δbcp-2 Δ*BDAG_00967 mutant containing disruption in BDAG_01006 (*Δ*00967 01006^—^). (D to F) Interbacterial competition assays between Δ*bcp-1* donor bacteria (D), Δ*bcp-2* donor bacteria (E), or wild-type donor bacteria (F), competed against the indicated recipients: Δ*bcp-1* Δ*bcp-2* (parent; open circles), *Δbcp-1 Δbcp-2* recipients complemented with individual or both cognate *bcpI* genes (+*bcpI-1*, +*bcpI-2*, or +*bcpI-1*+*bcpI-2*; gray circles), *Δbcp-1 Δbcp-2* containing disruption mutations in BDAG_01005 (01005^—^, open triangles) or BDAG_01006 (01006^—^, open triangles), and Δ*bcp-1 Δbcp-2* ΔBDAG_00967 mutant (ΔBDAG_00967, open squares) or *Δbcp-1 Δbcp-2 Δ*BDAG_00967 mutant containing disruption in BDAG_01006 (Δ00967 01006^—^, gray circles). Symbols represent log_10_ competitive index values (ratio of donor to recipient) from three independent experiments and bars show the mean (*n* = 8 to 9). Dashed line shows log_10_ competitive index = 0; ns; not significant; *, *P < *0.05.

The contributions of three of the four candidate genes (*cspD*, *hisD*, and BDAG_00967) did not match the competitive phenotypes of their respective CDI-resistant transposon mutants (Fig. 2). To determine whether these transposon mutants harbored additional mutations that impacted CDI susceptibility, whole-genome sequencing was performed. Reads were mapped to the *B. dolosa* AU0158 reference genome and compared to sequencing performed in parallel on the parental Δ*bcp-1* Δ*bcp-2* mutant. In addition to the expected Δ*bcp-1* and Δ*bcp-2* deletions, the parent mutant (and derived Tn mutants) contained several variations compared to the published reference genome (Table S6). Each transposon mutant also had at least one unique mutation in its genome (Table 1). Strikingly, the three transposon mutants suspected of harboring additional mutations all contained mutations within a single locus predicted to encode LPS biosynthesis proteins (Fig. 4A). Tn2-2 (*hisD*:miniTn5) contained a mutation in BDAG_01005, encoding a predicted O antigen ligase. Tn1-2 (BDAG_00967:miniTn5) and Tn2-7 (*cspD*:miniTn5) each contained distinct mutations in BDAG_01006, which encodes a putative glycosyltransferase. Notably, Tn2-7 (*cspD*::miniTn5) also contained a frameshift mutation in BDAG_00967. Consequently, Tn2-7 and Tn1-2 each contained independent mutations in both BDAG_00967 and BDAG_01006.

**TABLE 1:** Unique variations^*a*^ in *B. dolosa* transposon mutants identified by whole-genome resequencing^*b*^

Tn mutant^*c*^	Chr	Position	Variation^*d*^	Locus tag	Annotation	Cov.^*e*^	Freq. (%)^*f*^
Tn1-2 (BDAG_00967)	1	860,238	Δ17 bp^*g*^	BDAG_01006 (AK34_796)	Glycosyl transferase	88	100
Tn1-2 (BDAG_00967)	2	850,831	C > G [A→G]	BDAG_03419 (AK34_3910)	Nitrate/sulfite reductase	109	100
Tn2-2 (*hisD*)	1	859,798	(AGC) 4→3 [Δ L]	BDAG_01005 (AK34_795)	O-antigen ligase	255	99.8
Tn2-7 (*cspD*)	1	810,641	Δ1 bp^*g*^	BDAG_00967 (AK34_755)	EAL domain-containing protein	115	100
Tn2-7 (*cspD*)	1	860,374	A > G [L→P]	BDAG_01006 (AK34_796)	Glycosyl transferase	127	100
Tn2-8 (*cepR*)	3	562,405	A > T [L→M]	BDAG_04624 (AK34_5528)	MFS transporter	218	100

a Variations relative to AU0158 reference genome occurring at >85% frequency in regions having >25 mapped reads that were identified in the indicated transposon mutant and absent from Δ*bcp-1* Δ*bcp-2* parent mutant.

b Chr, chromosome; Cov, coverage; Freq, frequency; MFS, major facilitator superfamily.

c Parenthesis denote transposon-disrupted gene.

d Brackets show amino acid change, where applicable.

e Number of mapped reads.

f Percent reads containing indicated mutation.

g Mutations causing ORF frameshift.

The Tn2-8 (*cepR*:miniTn5) mutant was found to have a single unique mutation in gene BDAG_04624, encoding a putative major facilitator super family sugar transporter. Disruption of BDAG_04624 by integration of a suicide plasmid within its coding sequence did not affect recipient cell susceptibility to CDI system-1 or-2 (Fig. 1A; Fig. S2). Although we cannot rule out a possible contribution of the precise amino acid change (Leu to Met) found in Tn2-8, these results are consistent with the finding that the *cepR*::miniTn5 transposon mutant and Δ*cepR* mutant showed similar CDI sensitivities and suggest that BDAG_04624 does not contribute significantly to recipient cell intoxication.

### Alterations to recipient cell LPS impacts susceptibility to *B. dolosa* CDI systems-1 and -2.

We hypothesized that alterations to LPS were responsible for the CDI resistance of transposon mutants Tn1-2, Tn2-2, and Tn2-7. To determine whether the mutations identified by whole-genome sequencing correlated with LPS changes, LPS banding patterns for wild-type and mutant *B. dolosa* were qualitatively examined. As expected, no differences were observed between wild-type bacteria, the Δ*bcp-1* Δ*bcp-2* mutant, and Tn2-8 (*cepR*:miniTn5) (Fig. 4B). In contrast, Tn2-2 (*hisD*:miniTn5), containing a mutation in BDAG_01005 (Fig. 4B), showed a decrease in the amount of O-antigen and an altered LPS core. Both transposon mutants containing mutations in BDAG_01006 (Tn1-2 and Tn2-7) also yielded LPS that appeared to contain a reduced amount of O-antigen and core oligosaccharide, although the changes were distinct from those seen in Tn2-2.

To assess the role of BDAG_01005 and BDAG_01006 in susceptibility to CDI, disruption mutants were created by integrating a suicide plasmid within each open reading frame (ORF) in the Δ*bcp-1* Δ*bcp-2* mutant background. Disruption mutation of BDAG_01005 or BDAG_01006 resulted in LPS changes that appeared similar to those identified in the corresponding transposon mutants (Fig. 4B). When competed against Δ*bcp-1* or Δ*bcp-2* donor bacteria, all strains carrying disruption mutations in BDAG_01005 or BDAG_01006 were resistant to interbacterial toxicity, indicating that intact LPS is required for recipient cell intoxication by both CDI system-1 and -2 (Fig. 4D and E). Similar results were observed for recipient cells competed against wild-type *B. dolosa* (Fig. 4F).

Because two transposon mutants contained unique mutations in both BDAG_01006 and BDAG_00967 (either by transposon insertion into BDAG_00967 in the case of Tn1-2 or an additional unique mutation in the case of Tn2-7), we also tested disruption of BDAG_01006 in ΔBDAG_00967 mutant recipient cells. This double mutant produced LPS banding similar to that of the single BDAG_01006 mutant (Fig. 4B) and was similarly resistant to intoxication by wild-type bacteria (Fig. 4F), suggesting that the combination of these two mutations does not alter CDI sensitivity.

Taken together, these results indicate that genes BDAG_01005 and BDAG_01006 are involved in *B. dolosa* LPS biosynthesis and are required for recipient cell susceptibility to both CDI system-1 and -2.

### *B. dolosa* Δ*wabO* mutants produce altered LPS and are CDI resistant.

**FIG 5:**
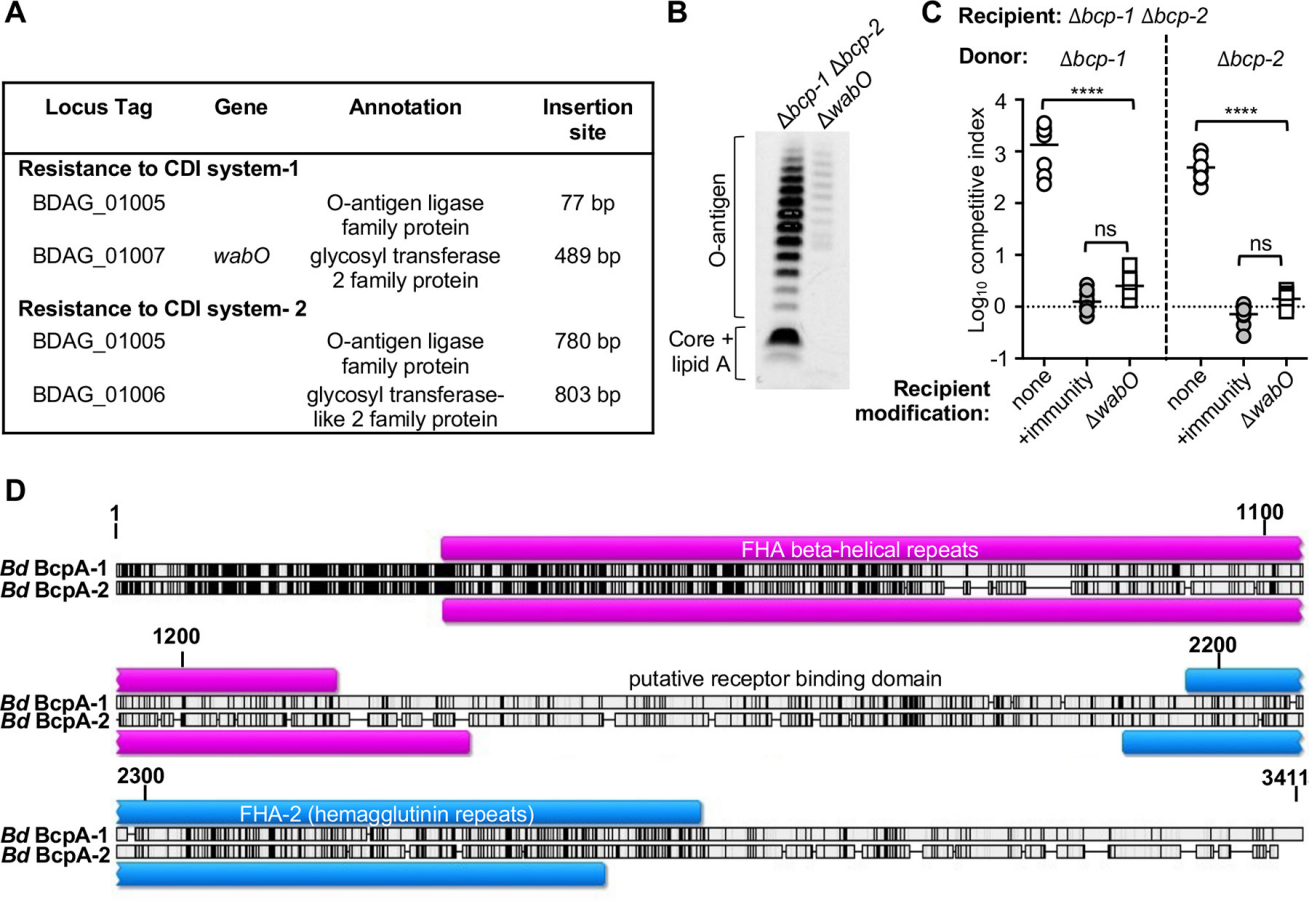
Role of *B. dolosa* recipient cell *wabO* to susceptibility to CDI systems-1 and -2. (A) Chromosomal insertion sites of CDI-resistant Δ*bcp-1* or Δ*bcp-2* recipient cells after serial competitions against wild-type *B. dolosa* donors. (B) Extracted LPS separated by SDS-PAGE and stained with Pro-Q Emerald 300 from Δ*bcp-1* Δ*bcp-2* mutant and Δ*bcp-1* Δ*bcp-2* Δ*wabO* (Δ*wabO*). (C) Interbacterial competition assays between Δ*bcp-1* (left) or Δ*bcp-2* (right) donor bacteria and the indicated recipients: Δ*bcp-1* Δ*bcp-2* (parent; open circles), Δ*bcp-1* Δ*bcp-2* complemented with cognate *bcpI* genes (+immunity, light gray circles) (*bcpI-1*, *bcp-2*, or *bcp-1*+*bcpI-2*) or Δ*bcp-1* Δ*bcp-2 ΔwabO* (Δ*wabO*, open squares). Symbols represent log_10_ competitive index values (ratio of donor to recipient) from three independent experiments and bars show the mean (*n *= 9). Dashed line shows log_10_ competitive index = 0; ns, not significant; ****, *P < *0.0001. (D) Schematic of amino acid alignment of *B. dolosa* AU0158 BcpA-1 (top line) and BcpA-2 (bottom line), showing location of FHA-1 (pink) and FHA-2 (blue) repeat regions. Shading indicates sequence conservation. Black, identical residues; gray, similar residues; white, no similarity.

Although our initial transposon selection approach yielded CDI-resistant clones, the results suggested that problems with population bottlenecks and/or low mutant diversity contributed to the selection of mutations that were independent of the transposon insertions. Considering these results, we repeated the mutagenesis and sequential interbacterial competition approach, identifying Δ*bcp-1* and Δ*bcp-2* recipient cell clones that were resistant to intoxication by wild-type *B. dolosa*. Among the identified CDI-resistant transposon mutants were clones having insertions in BDAG_01005, BDAG_01006, and the upstream gene BDAG_01007 (Fig. 5A). Protein BDAG_01007 (WabO) shares 80% amino acid identity (88% similarity) with the Burkholderia cenocepacia J2315 WabO homolog (BCAL2402), although the genes have different genomic contexts. A *B. dolosa* Δ*wabO* mutant produced LPS with reduced O antigen and a truncated core region (Fig. 5B), consistent with previous findings of B. cenocepacia *wabO* mutants (23). The Δ*wabO* mutant was also resistant to intoxication by CDI system-1 and -2 (Fig. 5C), providing further support for the role of this three gene locus in recipient cell sensitivity to *B. dolosa* BcpA-1 and BcpA-2.

### Putative receptor binding domains of *B. dolosa* BcpA-1 and BcpA-2 are distinct.

These data indicate that alterations to recipient cell LPS impact intoxication by two distinct BcpA proteins, likely acting at the BcpA-CT effector entry steps of the pathway. Results from E. coli CdiA proteins have shown that the outer membrane receptor binding domain corresponds to a central region of the protein, between the FHA-1 and FHA-2 repeat regions (9, 11). CdiA/BcpA proteins that utilize the same OM receptor would be expected to share sequences within the receptor binding domain. The *B. dolosa* BcpA-1 and BcpA-2 proteins are quite different, having 35% amino acid identity overall (Fig. 5D). Moreover, while the predicted FHA-1 and FHA-2 regions are 39% and 34% identical, respectively, the regions containing the putative receptor binding domain are only 24% identical. Within this larger dissimilar central domain, however, localized regions of slightly higher conservation can be found. Future work is needed to delineate whether these two distinct BcpA proteins utilize the same outer membrane receptor (perhaps via a locally conserved region of the putative receptor binding domain) or whether their distinct receptors were simply both impacted by the LPS disruptions that were tested here.

## DISCUSSION

*Burkholderia* species, including *B. dolosa*, represent effective models for examining the molecular mechanisms controlling contact-dependent growth inhibition (CDI) systems. As it deploys two distinct CDI systems under laboratory conditions, *B. dolosa* AU0158 can be used to identify both specific and general mechanisms that impact CDI. In this study, we used transposon mutagenesis to identify a limited number of factors required in *B. dolosa* recipient cells for susceptibility to each unique CDI system. Follow up studies demonstrated that disruption to regulatory networks marginally impacted *B. dolosa* competitive fitness, while alterations to LPS enabled nonimmune recipient cells to resist intoxication by two distinct CDI systems.

As studies that identify CDI susceptibility factors in *Burkholderia* spp. (β-proteobacteria) have been limited, our understanding of CDI system effector import has been primarily guided by findings in E. coli and other γ-proteobacteria (10, 24). According to the current model, intoxication via the CDI system protein BcpA requires both extracellular and intracellular proteins to facilitate translocation into the recipient cell cytoplasm. Inner membrane proteins GltJK and Bth_II0599 have been found to facilitate translocation of BcpA-CT from *B. multivorans* and B. pseudomallei, respectively (15, 16). This study has not identified candidate inner membrane receptors for *B. dolosa* BcpA-1 or BcpA-2, potentially due to a lack of saturation in the mutagenesis scheme or essentiality of the receptor encoding genes. The BcpA-CT regions of these two proteins, which includes the putative inner membrane translocation domain, share only 16% amino acid identity, suggesting that they require distinct inner membrane proteins for recipient cell entry.

Our data also suggest that alterations to recipient cell regulatory pathways can impact interbacterial competition efficiency. In B. thailandensis, the acyl-homoserine lactone (AHL)-based quorum sensing (QS) system BtaIR1 is required for *bcpAIOB* expression and for donor cells to outcompete nonimmune recipient bacteria by CDI (20). While it is not known whether CepI/CepR similarly influence *bcpAIOB* expression in *B. dolosa*, here we show that CepR also impacts CDI in recipient cells via an unknown, likely indirect, mechanism. LPS does not appear to be affected by loss of *cepR*, as the *cepR*::miniTn5 mutant produced LPS similar to wild-type *B. dolosa* (Fig. 4B). The CepI/CepR QS system, which has been well-characterized in several members of the B. cepacia complex (25), is modulated by additional regulators and its regulon overlaps with those of other QS systems (26–28). It is unclear why *cepI* mutant recipient cells behaved differently than *cepR* mutants in our competition assays, although there is evidence in other species that some LuxR-type receptors can respond to multiple autoinducers (29).

Our data also indicate that, in the absence of *bcp-1* and *bcp-2*, recipient cells lacking *cepR* have a CDI-independent competitive advantage against *cepR*+ bacteria on agar surfaces. Although B. thailandensis *btaR1* mutants have a fitness advantage in liquid culture, QS-controlled type 6 secretion system activity prevents these QS mutants from outcompeting wild-type bacteria on solid agar (21). Our results suggest that such policing of QS mutants does not occur in the same manner for *B. dolosa*. In our interbacterial competition assay, it is plausible that a small growth or fitness advantage for one strain could skew the spatial distribution of donor and recipient cells, which would impact the frequency of antagonistic interactions and, thus, competition outcome. Unraveling possible CDI-dependent or -independent mechanisms that improve the competitive fitness of Δ*cepR* recipient cells will provide insight into the interplay of these distinct mechanisms of interbacterial communication.

Strikingly, independent genetic evidence showed that mutations within three genes, *wabO*, BDAG_01006, and BDAG_01005, conferred resistance to *B. dolosa* BcpA-1 and BcpA-2. In the first mutagenesis experiment, all three transposon mutants whose CDI resistance could not be attributed to the transposon insertion (*hisD*::miniTn5, *cspD*::miniTn5, and BDAG_00967::miniTn5) harbored additional mutations within this locus. Reselection of new transposon mutants also identified these three genes as candidate susceptibility factors. LPS biosynthesis is a multistep process requiring multiple genes, so it is unclear why independent selections repeatedly identified only these three genes. Overall fitness and growth rate of LPS mutants may have contributed, as any mutants with decreased growth rates would likely be eliminated in our selection approach.

While outer membrane proteins have been primarily identified as E. coli CdiA receptors (11, 13, 24), thus far, LPS is the only recipient cell surface molecule identified to participate in *Burkholderia* CDI. Similar to our results, disruption of B. thailandensis LPS by mutation of Bth_I0986 was found to prevent entry of a B. pseudomallei BcpA toxin (16). B. thailandensis Bth_I0986 does not show significant amino acid identity to *wabO*, BDAG_01005 or BDAG_01006. Unlike mutation of these *B. dolosa* LPS biosynthesis genes, which conferred resistance to two distinct CDI systems, mutation of Bth_I0986 resulted in specific resistance to a B. pseudomallei 1026b BcpA, but not B. thailandensis E264 BcpA (16). Bacteria producing truncated LPS often display pleiotropic effects, including altered membrane properties and elevated membrane stress responses (30, 31), which could plausibly impact BcpA-CT import into recipient bacteria indirectly. Alternatively, BcpA could interact directly with recipient cell LPS to facilitate toxin entry, as is the case for LPS-binding phages and bacteriocins (32–34). Very recent evidence indicates that a subclass of CdiA proteins utilizes E. coli LPS inner core polysaccharides as recipient cell receptors (35). Our results suggest that related mechanisms may be found in diverse CDI systems.

Although our work showed that intoxication by both BcpA-1 and BcpA-2 were similarly impacted by major alterations to LPS, it is unclear whether the proteins utilize the same recipient cell outer membrane receptor. The BDAG_01006, BDAG_01005, and *wabO* mutations tested here dramatically altered the LPS profiles, potentially disrupting multiple distinct polysaccharide structures that could each serve as a receptor for different BcpA proteins. Alternatively, it is possible that, although BcpA-1 and BcpA-2 share little sequence identity overall, a small region of homology within the putative receptor binding domain is responsible for mediating interactions with a single outer membrane receptor.

*Burkholderia* strains displaying diverse LPS and O-antigen phenotypes have been isolated from clinical and environmental settings (36–39). The natural niches in which *Burkholderia* CDI systems are active or provide fitness advantages are not known, but our results suggest that strains producing truncated LPS may be insensitive to BcpA. Future delineation of the molecular mechanisms underlying the role of LPS in *Burkholderia* CDI and the consequences of these interactions will yield important insight into the contribution of CDI systems to bacterial communication and antagonism.

## MATERIALS AND METHODS

### Bacterial strains and culture conditions.

Burkholderia dolosa AU0158 strains used in this study are listed in Table S1 and were cultured in low-salt (0.5% NaCl) Luria-Bertani medium (LSLB). Plasmids were maintained in Escherichia coli DH5α and delivered to *B. dolosa* using conjugation donor strain E. coli RHO3, a 2,6-diaminopimelic acid (DAP) auxotroph (40). For selection of *B. dolosa*, LSLB was supplemented with 250 to 500 μg/ml kanamycin or 50 to 125 μg/ml tetracycline. E. coli strains were cultured in LSLB supplemented, where appropriate, with 100 μg/ml ampicillin, 50 μg/ml kanamycin, 25 μg/ml tetracycline, or 200 μg/ml DAP.

### Genetic manipulations.

Plasmids used are listed in Table S2. All plasmids were confirmed by DNA sequencing (Eurofins Genomics or ACGT, Inc.), and bacterial mutant strains were verified by PCR. Complete locus tag and accession number information for all analyzed genes are available in Table S3.

In-frame deletion mutations were constructed by allelic exchange using plasmid pEXKm5 (40). Plasmids for gene deletions were constructed by PCR amplification of two fragments: one fragment ~500 bp 5′ to the ORF (including the first 3 to 5 codons) and another ~500 bp 3′ to the ORF (including the last 3 to 15 codons). For deletion of *cspD*, *cepR*, *cepI*, and *wabO*, these fragments were joined by overlap PCR and cloned into pEXKm5 by restriction digestion, resulting in plasmids pΔcspD_overlap, pECG110, pECG111, and pTMM060, respectively. For deletion of BDAG_00967 and *hisD*, Gibson assembly using Gibson Assembly HiFi 1 Step (Synthetic Genomics Inc.) reagents were used to join the two PCR fragments with linearized pEXKm5, resulting in plasmids pΔBDAG_00967_overlap and pΔhisD_overlap, respectively.

Bacterial mutants were marked with antibiotic resistance cassettes by delivering pUC18Tmini-Tn-Kan (41) or pUCTet (5) to an *att*Tn7 site within the AU0158 genome. Markers were delivered via triparental matings of E. coli RHO3 with helper plasmid pTNS3, as described previously (42). Successful delivery to an *att*Tn7 site associated with *glmS-1* (AK34_RS13635) or *glmS-3* (AK34_RS08675) was confirmed by PCR using primers Tn7L Fw (43) and glmS1 Rev or glmS3 Rev (7) (Table S4).

To complement AU0158 deletion and transposon mutants, the gene of interest was PCR amplified and cloned into an *att*Tn7 site delivery plasmid. For *hisD*, BDAG_00967, *cepR*, and *cepI*, ORFs were cloned by restriction digestion into pUCS12 (5), 3′ to the strong, constitutive promoter P_S12_ (Burkholderia thailandensis E264 *rpsL* gene promoter), resulting in plasmids pS12-*hisD*, pS12-00967, pS12-*cepR*, and pS12-*cepI*. For complementation of kanamycin-resistant miniTn5 mutants, BDAG_00967, BDAG_00966, and a fragment containing both BDAG_00966 and BDAG_00967 in their native orientation were PCR-amplified and cloned 3′ to P_S12_ in the tetracycline-resistant backbone, pUCTet (5), resulting in plasmids pS12-00967-Tet, pS12-00966-Tet, and pS12-00967-66-Tet. All genes for complementation were delivered to *att*Tn7 sites in the AU0158 genome via triparental mating with helper plasmid pTNS3 as described previously (42).

To construct the *cepI* beta-galactosidase reporter, the 208-bp intergenic region located 5′ to the *cepI* ORF was PCR-amplified and cloned upstream of the promoterless *lacZ* in the *att*Tn7 site delivery plasmid, pUClacZ (5), resulting in plasmid pECG112. The reporter was delivered to an *att*Tn7 site in the genome of double mutant Δ*bcp-1* Δ*bcp-2* and triple mutants Δ*bcp-1* Δ*bcp-2* Δ*cepR* and Δ*bcp-1* Δ*bcp-2* Δ*cepI* as described above.

To generate disruption mutations by plasmid integration, ~500 bp of internal sequence of the ORF were PCR-amplified (primers listed in Table S4), corresponding to 158 to 675 bp of BDAG_01005 (of 1281 total bp), 26 to 573 bp of BDAG_01006 (of 843 total bp), and 86 to 629 bp of BDAG_04624 (of 1,209 bp total). Fragments were cloned into the multiple cloning site of pUC18-miniTn5-Km, resulting in plasmids pECG113, pECG114, and pECG115. Suicide plasmids were mated into AU0158 Δ*bcp-1* Δ*bcp-2* in the absence of pTNS3 (preventing *att*Tn7 site delivery) and integration of the appropriate plasmid into chromosomal BDAG_01005, BDAG_01006, or BDAG_04624 was confirmed by PCR.

For strains overexpressing *bcpAIOB-1* and *bcpAIOB-2*, promoter P_S12_ was excised from plasmid pUCS12 (5) by restriction digestion and cloned 5′ to the first ~500 nucleotides of *bcpA-1* or *bcpA-2* (PCR-amplified) into pUC18-miniTn7T-kan (41), resulting in plasmids pS12AP6 and pS12AP7, respectively. These plasmids were mated into AU0158 Δ*bcp-1* and Δ*bcp-2* without inclusion of the helper plasmid pTNS3 (to prevent *att*Tn7 site delivery). Kanamycin-resistant colonies that carried pS12AP6 or pS12AP7 cointegrated 5′ to *bcpAIOB-1* or *bcpAIOB-2* were obtained and confirmed by PCR, resulting in the positioning of P_S12_ immediately 5′ to the chromosomal copy of each *bcp* locus, similar to previously described strains (7). These strains (Δ*bcp-1* P_S12_-*bcp-2* and Δ*bcp-2* P_S12_-*bcp-1*) were routinely cultured with kanamycin to select for plasmid retention.

### Transposon mutagenesis selection.

Random transposon mutagenesis of the AU0158 Δ*bcp-1* Δ*bcp-2* double mutant or Δ*bcp-1* and Δ*bcp-2* single mutants was conducted by delivering pUT-miniTn5-Kn (15, 44) by conjugation. The mating was collected, serially diluted in PBS, and plated on LSLB with 250 μg/ml kanamycin to select for transposon insertion mutants. Isolated colonies were pooled in LSLB with 15% glycerol for storage of the transposon mutant library at −80°C.

For selection of CDI resistant mutants, sequential interbacterial competition assays were used (15). In brief, 25 μL of the transposon pool was inoculated into 25 ml LSLB with 250 μg/ml kanamycin and cultured overnight. Tetracycline-resistant donor strains (Δ*bcp-1*, Δ*bcp-2*, or wild type) were cultured overnight in LSLB with 50 μg/ml tetracycline. Cultures were pelleted via centrifugation at 15,000 × *g* for 5 min and resuspended in sterile PBS to optical deinsity at 600 nm (OD_600_) = 2. Interbacterial competition assays were performed as described below with the transposon pool (recipients) mixed 1:1 with the donor strain. Input ratios were determined by serially diluting the initial mixture and plating dilutions on antibiotic plates. For competitions, 20 μl of the mixture was spotted on LSLB agar (without antibiotics) in triplicate and incubated at 37°C for 48 h. Cocultures were collected with a sterile loop, serially diluted in sterile PBS and plated on antibiotic plates to determine the output competition ratios. Kanamycin-resistant recipient colonies were collected from output plates and pooled from all replicates in LSLB with 15% glycerol for storage. This output pool of transposon mutants was used to inoculate fresh LSLB (25 ml) and was recompeted against the appropriate donor strain in the next round of selection. In total, three rounds of competition selection were performed.

### Arbitrary PCR.

Transposon insertion sites of CDI-resistant mutants were determined using arbitrary PCR, as described previously (15). Genomic DNA was extracted from transposon mutants and wild-type AU0158 strain (as a control) using the Wizard Genomic DNA Purification system (Promega) according to the manufacturer’s protocol. Nested arbitrary-primed PCR was performed using this genomic DNA as template with primers Arb1 (arbitrary primer) and Tn3out (first round primer annealing to the 3′ end of the transposon) (Table S4). PCR products were treated with ExoSAP-IT PCR Product Cleanup Reagent (Applied Biosystems) or ExoProStar (Cytiva) and used as templates for the second, nested PCR with primers Arb2 and Tn3in. Second-round PCR products from transposon mutants were compared to the wild-type AU0158 negative control by agarose gel electrophoresis and treated with ExoSAP-IT or ExoProStar. The transposon-chromosome junctions in the second-round PCR products were sequenced with primer Tn3seq and transposon-disrupted genes identified by BLAST analysis.

### Interbacterial competition assay.

Interbacterial competition assays were performed as previously described (5, 7) with modifications. *B. dolosa* strains carrying antibiotic resistance cassettes at *att*Tn7 sites were cultured overnight without antibiotics and resuspended in sterile PBS to OD_600_ = 2. Unless otherwise indicated, bacteria were mixed at a 1:1 ratio, 5 or 20 μl of the mixture was plated on LSLB agar in triplicate, and plates were incubated at 37°C for 24 or 26 h. The input ratio (donor:recipient) was determined by plating the coculture inoculum on antibiotic plates. Bacteria were collected from cocultures with a sterile loop, diluted in sterile PBS, and plated on LSLB with antibiotic selection to quantify each strain. The competitive index (CI) was calculated as the ratio of the donor strain to the recipient strain at 24 h divided by the input (donor:recipient) ratio. For bacterial competitions between tetracycline-resistant donor strains and recipient strains resistant to both kanamycin and tetracycline (Kan^R^ Tn mutants complemented with Tet^R^ constructs), donor strain CFU were calculated by subtracting the recipient (kanamycin-resistant) CFU from the total (tetracycline-resistant) CFU prior to CI calculation. At least three independent experiments were performed in triplicate.

### Beta-galactosidase assay.

Triplicate 2-ml cultures of reporter strains were incubated overnight at 37°C with aeration in LSLB. For samples supplemented with AHL, *N*-octanoyl-l-homoserine lactone (C8-HSL; Sigma) was added at 2 μM. Beta-galactosidase assays were performed as described previously (45), using a SpectraMax 5M plate reader (Molecular Devices). Two independent experiments were performed, each with three biological replicates.

### Whole-genome resequencing.

Genomic DNA was extracted from transposon mutants and AU0158 Δ*bcp-1* Δ*bcp-2* (as a control) using the Wizard Genomic DNA Purification system (Promega) according to the manufacturer’s protocol. Whole-genome resequencing and variant analysis were performed by ACGT, Inc. (Wheeling, IL) and Microbial Genome Sequencing Center, LLC (Pittsburgh, PA). In brief, DNA sequencing libraries were constructed as per manufacturer’s instructions (Illumina). The libraries were bar-coded with index tags. Libraries were sequenced using Illumina NextSeq 200 systems (for Microbial Genome Sequencing Center) or Illumina NextSeq 500 systems (for ACGT) to generate reads. Reads were mapped against the publicly available Burkholderia dolosa AU0158 genome (NCBI).

### LPS extraction and analysis.

Cells were cultured overnight in LSLB medium, normalized to OD_600_=2.0, and lipopolysaccharide (LPS) was extracted from a 1-ml culture using an LPS Extraction kit (iNtRON Biotechnology), per manufacturer's instructions. Extracted LPS was resuspended in SDS-PAGE sample buffer and equal volumes analyzed by SDS-PAGE on Novex 10–20% Tricine Gels (Invitrogen). LPS bands were visualized using Pro-Q Emerald 300 LPS Gel Stain (Invitrogen), per manufacturer’s instructions and images captured on a Gel Doc EZ Imager (Bio-Rad).

### Statistics and bioinformatics.

Data were analyzed by one-way ANOVA with Tukey *post hoc* tests using the statistical package in GraphPad Prism (v.8). Protein alignment was performed using the Clustal W alignment feature of Geneious R6 (v6.1.8). Domain predictions were performed using NCBI Conserved Domain search.
