# Supplementary material for: Recipient Cell Factors Influence Interbacterial Competition Mediated by Two Distinct Burkholderia dolosa Contact-Dependent Growth Inhibition Systems
Source: J Bacteriol. 2022 Aug 24;204(9):e00541-21. doi: 10.1128/jb.00541-21 (PMC9487645; doi:10.1128/jb.00541-21)
Supplement: Supplemental file 1 — Tables S1 to S7 and Fig. S1 to S3. Download jb.00541-21-s0001.pdf, PDF file, 1.7 MB [file jb.00541-21-s0001.pdf]

SUPPLEMENTAL INFORMATION

Recipient cell factors influence interbacterial competition mediated by two distinct *Burkholderia dolosa* contact-dependent growth inhibition systems

Zaria K. Elery*, A. Elizabeth Oates*, Tanya Myers-Morales, and Erin C. Garcia

Department of Microbiology, Immunology, and Molecular Genetics, University of Kentucky, Lexington, KY 40536

SUPPLEMENTAL INFORMATION

CONTENT LIST

SUPPLEMENTAL FIGURES

Fig. S1. Beta-galactosidase activity of a P_{cepI}-*lacZ* reporter in Δ*cepR* and Δ*cepI* mutant recipient cells.

Fig. S2. Role of recipient cell *cepR and cepI* during CDI system 2-mediated interbacterial competition.

Fig. S3. Growth of *B. dolosa hisD* mutants in minimal medium.

SUPPLEMENTAL TABLES

Table S1. *Burkholderia* strains used in this study.

Table S2. Plasmids used in this study.

Table S3. Gene name and accession number information.

Table S4. Oligonucleotide primers used in this study for arbitrary PCR, confirmation of *att*Tn7 site delivery, and construction of disruption mutants.

Table S5. Chromosomal insertion sites of CDI-resistant *B. dolosa* miniTn5 mutants.

Table S6. Variations in *B. dolosa* Δ*bcpAIOB-1* Δ*bcpAIOB-2* mutant identified by whole genome re-sequencing.

Table S7. Raw data (log_{10} competitive index values or Miller units) for all figures.

Table S1. ***Burkholderia dolosa*** **strains used in this study.**

Strain	Description	Reference
B. dolosa AU0158	Wild-type strain	
Δ*bcp-1*	Δ*bcpAIOB-1*	(1)
Δ*bcp-2*	Δ*bcpAIOB-2*	(1)
Δ*bcp-1* Δ*bcp-2*	Δ*bcpAIOB-1* Δ*bcpAIOB-2*	(1)
Δ*bcp-1* Kan	Δ*bcpAIOB-1 att*Tn7::GFP-Kan	This study
Δ*bcp-2* Kan	Δ*bcpAIOB-2att*Tn7::Kan	(1)
Δ*bcp-1* Δ*bcp-2* Kan	Δ*bcpAIOB-1* Δ*bcpAIOB*-2 *att*Tn7::Kan	This study
Δ*bcp-1* Tet	Δ*bcpAIOB-1 att*Tn7::Tet	(1)
Δ*bcp-2* Tet	Δ*bcpAIOB-2att*Tn7::Tet	(1)
Δ*bcp-1* Δ*bcp-2* +*bcpI-1*	Δ*bcpAIOB-1* Δ*bcpAIOB-2 att*Tn7::pAP3	This study
Δ*bcp-1* Δ*bcp-2* +*bcpI-2*	Δ*bcpAIOB-1* Δ*bcpAIOB-2 att*Tn7::pAP5	This study
Δ*bcp-1* Δ*bcp-2* +*bcpI-1* +*bcpI-2*	Δ*bcpAIOB-1* Δ*bcpAIOB-2 att*Tn7::pAP3 *att*Tn7::pAP5	This study
Δ*bcp-1* Δ*bcp-2* Tet	Δ*bcpAIOB-1* Δ*bcpAIOB-2 att*Tn7::Tet	This study
Δ*bcp-1* P_{S12}-*bcp-2*	Δ*bcp-1*::pS12AP7; replaces native *bcpA-2* promoter with P_{S12} to overexpress *bcpAIOB-2*	This study
Δ*bcp-2* P_{S12}-*bcp-1*	Δ*bcp-2*::pS12AP6, replaces native *bcpA-1* promoter with P_{S12} to overexpress *bcpAIOB-1*	This study
Δ*bcp-1* Δ*bcp-2* Δ*cspD*	Δ*bcpAIOB-1* Δ*bcpAIOB-2* ΔBDAG_02644	This study
Δ*bcp-1* Δ*bcp-2* Δ*hisD*	Δ*bcpAIOB-1* Δ*bcpAIOB-2* ΔBDAG_02714	This study

Δ*bcp-1* Δ*bcp-2* Δ*cspD* Kan	Δ*bcpAIOB-1* Δ*bcpAIOB-2* ΔBDAG_02644 *att*Tn7::Kan	This study
Δ*bcp-1* Δ*bcp-2* Δ*hisD* Kan	Δ*bcpAIOB-1* Δ*bcpAIOB-2* ΔBDAG_02714 *att*Tn7::Kan	This study
Δ*bcp-1* Δ*bcp-2* Δ*hisD* +*hisD*	Δ*bcpAIOB-1* Δ*bcpAIOB-2* ΔBDAG_02714 *att*Tn7::pS12-*hisD*	This study
Δ*bcp-1* Δ*bcp-2* +*hisD*	Δ*bcpAIOB-1* Δ*bcpAIOB-2* *att*Tn7::pS12-*hisD*	This study
Δ*bcp-1* Δ*bcp-2* Δ00967	Δ*bcpAIOB-1* Δ*bcpAIOB-2* ΔBDAG_00967	This study
Δ*bcp-1* Δ*bcp-2* Δ00967 +*gfp*	Δ*bcpAIOB-1* Δ*bcpAIOB-2* ΔBDAG_00967 *att*Tn7::GFP-Kan	This study
Δ*bcp-1* Δ*bcp-2* Δ00967 +00967	Δ*bcpAIOB-1* Δ*bcpAIOB-2* ΔBDAG_00967 *att*Tn7::pS12-00967-Kan	This study
Δ*bcp-1* Δ*bcp-2* Kan Tet	Δ*bcpAIOB-1* Δ*bcpAIOB-2* *att*Tn7::Kan *att*Tn7::Tet	This study
Δ*bcp-1* Δ*bcp-2* +*bcpI*-1 Tet	Δ*bcpAIOB-1* Δ*bcpAIOB-2* *att*Tn7::pAP3 *att*Tn7::Tet	This study
Δ*bcp-1* Δ*bcp-2* +*bcpI*-2 Tet	Δ*bcpAIOB-1* Δ*bcpAIOB-2* *att*Tn7::pAP5 *att*Tn7::Tet	This study
Δ*bcp-1* Δ*bcp-2* Tn1-2 +00967	miniTn5 transposon mutant Tn1-2 (Δ*bcpAIOB-1* Δ*bcpAIOB-2*) *att*Tn7::pS12-00967-Tet	This study
Δ*bcp-1* Δ*bcp-2* Tn1-2 +00966	miniTn5 transposon mutant Tn1-2 (Δ*bcpAIOB-1* Δ*bcpAIOB-2*) *att*Tn7::pS12-00966-Tet	This study
Δ*bcp-1* Δ*bcp-2* Tn1-2 +00967-66	miniTn5 transposon mutant Tn1-2 (Δ*bcpAIOB-1* Δ*bcpAIOB-2*) *att*Tn7:: pS12-00967-66-Tet	This study
Δ*bcp-1* Δ*bcp-2* Tn1-2 Tet	miniTn5 transposon mutant Tn1-2 (Δ*bcpAIOB-1* Δ*bcpAIOB-2*) *att*Tn7::Tet	This study
Δ*bcp-1* Δ*bcp-2* Δ*cepR*	Δ*bcpAIOB-1* Δ*bcpAIOB-2* ΔBDAG_03544	This study
Δ*bcp-1* Δ*bcp-2* Δ*cepR* Kan	Δ*bcpAIOB-1* Δ*bcpAIOB-2* ΔBDAG_03544 *att*Tn7::Kan	This study

Δ*bcp-1* Δ*bcp-2* Δ*cepR* +*cepR*	Δ*bcpAIOB-1* Δ*bcpAIOB-2* ΔBDAG_03544 *att*Tn7::pS12-*cepR*	This study
Δ*bcp-1* Δ*bcp-2* Δ*cepI*	Δ*bcpAIOB-1* Δ*bcpAIOB-2* ΔBDAG_03546	This study
Δ*bcp-1* Δ*bcp-2* Δ*cepI* Kan	Δ*bcpAIOB-1* Δ*bcpAIOB-2* ΔBDAG_03546 *att*Tn7::Kan	This study
Δ*bcp-1* Δ*bcp-2* Δ*cepI* +*cepI*	Δ*bcpAIOB-1* Δ*bcpAIOB-2* ΔBDAG_03546 *att*Tn7::pS12-*cepI*	This study
Δ*bcp-1* Δ*bcp-2* 01006⁻	Δ*bcpAIOB-1* Δ*bcpAIOB-2* BDAG_01006::pECG114 (disruption mutant)	This study
Δ*bcp-1* Δ*bcp-2* 01005⁻	Δ*bcpAIOB-1* Δ*bcpAIOB-2* BDAG_01005::pECG113 (disruption mutant)	This study
Δ*bcp-1* Δ*bcp-2* Δ00967 01006⁻	Δ*bcpAIOB-1* Δ*bcpAIOB-2* ΔBDAG_00967 BDAG_01005::pECG113 (disruption mutant)	This study
Δ*bcp-1* Δ*bcp-2* 04624⁻	Δ*bcpAIOB-1* Δ*bcpAIOB-2* BDAG_04624::pECG115 (disruption mutant)	This study
Δ*bcp-1* Δ*bcp-2* Δ*wabO*	Δ*bcpAIOB-1* Δ*bcpAIOB-2* ΔBDAG_01007	This study
Δ*bcp-1* Δ*bcp-2* Δ*wabO* Kan	Δ*bcpAIOB-1* Δ*bcpAIOB-2* ΔBDAG_01007 *att*Tn7::Kan	This study

Table S2. Plasmids used in this study.

Plasmid	Backbone	Description	Antibiotic resistance	Reference
pEXKm5		Allelic exchange vector	Kan	(2)
pTNS3		Helper plasmid for *att*Tn7 site delivery	Amp	(3)
pUC18Tmini-Tn7-km		*att*Tn7 site delivery vector (Kan-resistance cassette)	Amp, Kan	(4)
pUCTet	pUC18Tmini-Tn7-km	*att*Tn7 site delivery vector (Tet-resistance cassette)	Amp, Tet	(5)
pUT-miniTn5-Km	pGP704 derivative	Carries Tn5 transposase and mini-Tn5 transposon	Kan	(6)
pUC18mini-Tn*7*–kan–*gfp*		To deliver constitutive *gfp* gene to *att*Tn7 site	Kan	(7)
pUCS12	pUC18Tmini-Tn7-km	*att*Tn7 site delivery vector with P_{S12} promoter	Amp, Kan	(5)
pAP3	pUC18Tmini-Tn7-km	To deliver constitutively-expressed (P_{S12}) *bcpI-1* to *att*Tn7 site	Amp, Kan	(1)
pAP5	pUC18Tmini-Tn7-km	To deliver constitutively-expressed (P_{S12}) *bcpI-1* to *att*Tn7 site	Amp, Kan	(1)
pS12AP6	pUC18Tmini-Tn7-km	To integrate P_{S12} promoter 5' to *bcpAIOB-1*	Amp, Kan	This study
pS12AP7	pUC18Tmini-Tn7-km	To integrate P_{S12} promoter 5' to *bcpAIOB-2*	Amp, Kan	This study
p∆*cspD*_overlap	pEXKm5	To generate in-frame deletion of BDAG_02644	Kan	This study
p∆*hisD*_overlap	pEXKm5	To generate in-frame deletion of BDAG_02714	Kan	This study
pS12-*hisD*	pUC18Tmini-Tn7-km	To deliver constitutively-expressed (P_{S12}) BDAG_02714 to *att*Tn7 site	Amp, Kan	This study

p∆BDAG_00967_overlap	pEXKm5	To generate in-frame deletion of BDAG_00967	Kan	This study
pS12-00967-Kan	pUC18Tmini-Tn7-km	To deliver constitutively-expressed (P_{S12}) BDAG_00967 to *att*Tn7 site	Amp, Kan	This study
pS12-00967-Tet	pUCTet	To deliver constitutively-expressed (P_{S12}) BDAG_00967 to *att*Tn7 site	Amp, Tet	This study
pS12-00966-Tet	pUCTet	To deliver constitutively-expressed (P_{S12}) BDAG_00966 to *att*Tn7 site	Amp, Tet	This study
pS12-00967-66-Tet	pUCTet	To deliver constitutively-expressed (P_{S12}) BDAG_00967 and BDAG_00966 to *att*Tn7 site	Amp, Tet	This study
pECG110	pEXKm5	To generate in-frame deletion of *cepR*	Kan	This study
pECG111	pEXKm5	To generate in-frame deletion of *cepI*	Kan	This study
pS12-*cepR*	pUC18Tmini-Tn7-km	To deliver constitutively-expressed (P_{S12}) *cepR* to *att*Tn7 site	Amp, Kan	This study
pS12-*cepI*	pUC18Tmini-Tn7-km	To deliver constitutively-expressed (P_{S12}) *cepI* to *att*Tn7 site	Amp, Kan	This study
pECG10	pUC18Tmini-Tn7-km	To deliver P_{S12}-*lacZ* to *att*Tn7 site	Amp, Kan	(5)
pUClacZ	pUC18Tmini-Tn7-km	To deliver P_{neg}-*lacZ* (promoterless) to *att*Tn7 site	Amp, Kan	(5)
pECG112	pUC18Tmini-Tn7-km	To deliver P_{cepI}-*lacZ* to *att*Tn7 site	Amp, Kan	This study
pECG113	pUC18Tmini-Tn7-km	pD01005; ~500 bp internal fragment of BDAG_01005 for plasmid integration (disruption mutation)	Kan	This study

pECG114	pUC18Tmini-Tn7-km	pD01006; ~500 bp internal fragment of BDAG_01006 for plasmid integration (disruption mutation)	Kan	This study
pECG115	pUC18Tmini-Tn7-km	pD04624; ~500 bp internal fragment of BDAG_04624 for plasmid integration (disruption mutation)	Kan	This study
pTMM060	pEXKm5	To generate in-frame deletion of *wabO*	Kan	This study

Km, Kanamycin; Amp, Ampicillin; Tet, tetracycline

Table S3. Gene name and accession number information.

Gene name	NCBI gene locus tag	Old or previous locus tags		Protein accession no.
bcpA-1	AK34_RS22045	AK34_1816	BDAG_01897	WP_006764606
bcpI-1	AK34_RS29965	AK34_1815	N/A	WP_158380478
bcpA-2	AK34_RS06120	AK34_4390	BDAG_03003	WP_045552264
bcpI-2	AK34_RS06115	N/A	N/A	WP_035973792
--	AK34_RS16755	AK34_755	BDAG_00967	WP_045552424
--	AK34_RS16750	AK34_754	BDAG_00966	WP_006763745
hisD	AK34_RS26550	AK34_2721	BDAG_02714	WP_006765370
cspD	AK34_RS26155	AK34_2642	BDAG_02644	WP_006477070
cepR	AK34_RS03025	AK34_3773	BDAG_03544	WP_006766136
cepI	AK34_RS03015	AK34_3771	BDAG_03546	WP_006766138
--	AK34_RS11880	AK34_5528	BDAG_04624	WP_223297008
wabO	AK34_RS16960	AK34_797	BDAG_01007	WP_006763784
--	AK34_RS16955	AK34_796	BDAG_01006	WP_035971990
--	AK34_RS16950	AK34_795	BDAG_01005	WP_035971988

Table S4. Oligonucleotide primers used in this study for arbitrary PCR, confirmation of *att*Tn7 site delivery, and construction of disruption mutants.

Name	Sequence (5'-3')	Description	Reference
Tn3in	caagcgcgagatgttcaccgacc c	first round primer for arbitrary PCR, 3' end of miniTn5-kan transposon	(8)
Tn3out	ccacgcagatgggccggc	second round primer for arbitrary PCR, 3' end of miniTn5-kan transposon	(8)
Tn3seq	catcacacgaacaaagatgg	sequencing primer for 3' end of miniTn5-kan transposon	(8)
Arb1	ggccacgcgtcgactagtacnnn nnnnnnnacgcc	first round primer for arbitrary PCR	(8)
Arb2	ggccacgcgtcgactagtac	second round primer for arbitrary PCR	(8)
Tn7L Fw	attagcttacgacgctacaccc	anneals to miniTn7	(9)
glmS1 Rv	ttcggctgcgcgctc	reverse primer for *att*Tn7 site downstream of *glmS-1* in AU0158	(1)
glmS3 Rv	aaccggcatgtcgcgac	reverse primer for *att*Tn7 site downstream of *glmS-3* in AU0158	(1)
01005 F	GTCGCTgaattcATCTGC GCTCGCTCGTCAC	to amplify internal fragment of BDAG_01005 for disruption mutation (w/EcoRI site)	This study
01005 R	GTAGTAggtaccGAAACG CACGGCCACGAC	to amplify internal fragment of BDAG_01005 for disruption mutation (w/KpnI site)	This study
01006 F	GTCACTgaattcGGAACA ATCTGCCGTACC	to amplify internal fragment of BDAG_01006 for disruption mutation (w/EcoRI site)	This study
01006 R	GTAGTAggtaccCATCGA GAAGTCGTTGTCG	to amplify internal fragment of BDAG_01006 for disruption mutation (w/KpnI site)	This study

04624 F	GTCACTgaattcGCTTGTT GCCCGACATTG	to amplify internal fragment of BDAG_004624 for disruption mutation (w/EcoRI site)	This study
04624 R	GTAGTAggtaccAGC GGC ATC AAC GTA TGG	to amplify internal fragment of BDAG_004624 for disruption mutation (w/KpnI site)	This study

Table S5. Chromosomal insertion sites of CDI-resistant[a] *B. dolosa* miniTn5 mutants.

**Tn mutant[b]**	**Locus tag**	**Gene**	**Annotation**	**Insertion after gene bp**
Tn1-1, Tn1-2, Tn1-4, Tn1-6, Tn1-8, Tn2-5*	AK34_755 (BDAG_00967)		EAL domain-containing protein	224
Tn2-7*	AK34_2642 (BDAG_02644)	*cspD*	Cold-shock protein	35
Tn2-2*, Tn2-3*	AK34_2721 (BDAG_02714)	*hisD*	Histidinol dehydrogenase	373
Tn2-8*	AK34_3773 (BDAG_03544)	*cepR*	Autoinducer binding domain-containing protein	117

[a] Mutants isolated for resistance to *B. dolosa* BcpAIOB-2 are labeled "1-x". Mutants isolated for resistance to BcpAIOB-1 are labeled "2-x" and denoted with (*).

[b] Multiple transposon mutants are listed when sequencing results indicated they were clones with identical insertion sites.

Table S6. Variations[a] in *B. dolosa* Δ*bcpAIOB-1* Δ*bcpAIOB-2* mutant identified by whole genome re-sequencing.

					Seq run#1 [c]		Seq run#2 [c]		
**Ch**	**Position**	**Variation [b]**	**Locus tag**	**Annotation**	**Cov[d]**	**Freq (%)[e]**	**Cov[d]**	**Freq (%)[e]**	**Tn? [f]**
1	1,439,637	C→G [syn]	AK34_1335	acetyl-CoA acyl-transferase	306	92	38	100	Y
1	1,439,658	C→A [syn]	AK34_1335	acetyl-CoA acyl-transferase	339	88	25	100	Y
1	1,778,998	T→G [V→G]	AK34_1666	multidrug efflux transporter periplasmic subunit	294	99	132	100	Y
1	2,565,694	C→G	intergenic		ND	ND	55	96	N
2	453,761	T→G [V→G]	AK34_3567	enoyl-CoA hydratase	265	100	149	100	Y
2	612,089	$(TGGCAA)_{11\rightarrow12}$	intergenic		30	100	78	96	Y
2	614,406	$(ACTCAGG)_{2\rightarrow1}$	intergenic		25	100	154	99	Y
2	1,386,452	A→C	intergenic		145	100	96	100	Y
2	2,156,583	T→G	intergenic		122	100	124	100	Y

Ch, chromosome; Cov, coverage; Freq, frequency; ND, not detected

[a] Variations relative to AU0158 reference genome occurring at >85% frequency in regions having >25 mapped reads

[b] Brackets show amino acid substitution or synonymous change (syn), where applicable.

[c] Control Δ*bcpAIOB-1* Δ*bcpAIOB-2* parent strain was re-sequenced twice, with two batches of transposon mutants. Sequencing run #1 for comparison to transposon mutant Tn1-2 and sequencing run #2 for comparison to transposon mutants Tn2-2, Tn2-7, and Tn2-8.

[d] Number of mapped reads

[e] % reads containing indicated mutation

[f] Present (Y) in all re-sequenced transposon mutants. Variation denoted (N) was not detected in Tn1-2 or Tn2-8.

Table S7. Raw data (log_{10} competitive index values or Miller units) for all figures.

Figure

1A

Recipient	*Δbcp-1 Δbcp-2*	*+bcpI-1*	Tn 2-8	*ΔcepR*	04624-
Exp 1	2.08	0.32	1.26	1.14	2.32
	1.84	-0.09	0.69	0.74	2.26
	2.00	-0.08	1.46	1.04	2.36
Exp 2	2.07	0.62	1.300	0.63	1.97
	2.33	0.41	1.39	0.88	1.94
	2.06	0.33	1.35	1.25	1.78
Exp 3	2.72	0.20	1.76	1.87	2.73
	2.51	0.01	1.69	1.69	3.33
	2.49	-0.17	1.96	1.89	2.90

1B

Recipient	*Δbcp-1 Δbcp-2*	*+bcpI-1*	*ΔcepR*	*ΔcepR +cepR*
Exp 1	2.42	0.18	1.96	3.63
	3.08	-0.09	2.15	3.41
	2.95	-0.34	1.71	3.38
Exp 2	2.49	-0.40	2.00	3.50
	2.20	-0.50	1.73	3.30
	2.58	-0.40	2.07	3.14
Exp 3	2.80	-0.32	1.85	3.33
	2.66	-0.11	1.18	3.17
	2.47	-0.01	1.77	2.88

1C

Recipient	*Δbcp-1 Δbcp-2*	*+bcpI-1+bcpI-2*	*ΔcepR*	*ΔcepR +cepR*
Exp 1	4.26	-0.65	3.19	4.97
	2.94	-0.02	2.94	4.64
	5.08	0.23	2.65	4.76
Exp 2	3.49	0.34	1.39	3.22
	3.16	-0.14	1.60	3.75
	3.20	0.07	2.04	3.55
Exp 3	4.37	-0.42	3.42	6.50
	4.57	0.10	3.59	6.44
	4.68	0.03	3.70	6.58

1D

Recipient	*Δbcp-1 Δbcp-2*	*+bcpI-2*	*ΔcepI*	*ΔcepI +cepI*
Exp 1	1.51	-0.20	1.02	2.64
	1.21	-0.22	1.22	2.88
	1.29	-0.28	1.37	2.90
Exp 2	1.20	0.19	0.81	1.39
	1.32	0.27	0.89	1.42
	1.40	0.09	1.10	1.26
Exp 3	1.98	1.03	1.36	1.76
	1.85	-0.04	1.73	1.72
	1.88	-0.12	1.58	2.17

1E

Recipient	*Δbcp-1 Δbcp-2*	*+bcpI-2*	*ΔcepR*	*ΔcepR +cepR*
Exp 1	0.28	-0.10	-0.14	0.35
	-0.02	-0.20	-0.14	0.25
	0.20	0.00	-0.73	0.40
Exp 2	0.16	0.03	-0.20	-0.04
	0.02	-0.01	-0.73	0.12
	0.10	0.15	-0.65	-0.06
Exp 3	-0.13	0.05	-0.20	0.26
	-0.31	0.12	-0.29	0.33
	-0.23	-0.11	-0.07	0.20

2A

Recipient	*Δbcp-1 Δbcp-2*	*+bcpI-2*	Tn 2-2	*ΔhisD*	Tn 2-7	*ΔcspD*
Exp 1	2.57	0.06	1.34	3.66	-0.21	2.64
	2.66	0.43	0.51	3.64	-0.54	2.79
	2.53	0.22	0.96	3.53	-0.64	2.71
Exp 2	2.89	0.53	0.43	3.10	0.29	2.51
	2.53	0.22	0.87	2.99	-0.13	2.46
	2.74	0.14	0.29	3.02	0.01	2.44
Exp 3	2.93*	-0.08*	0.68	2.16	-0.06	2.51
	2.30*	0.25*	1.09	2.62	0.05	1.83
	2.04*	0.32*	0.49	2.22	-0.43	1.93

* Values are the same as in 2B Exp. 3 (first and second column) as both experiments (2A Expt. 3 and 2B Expt. 3) were performed at the same time, using these same controls.

2B

Recipient	*Δbcp-1 Δbcp-2*	*+bcpI-2*	Tn 1-2	Δ00967
Exp 1	2.84	-0.25	-0.29	2.32
	2.92	-0.23	-0.32	2.47
	2.92	-0.27	-0.21	2.54
Exp 2	3.06	0.51	0.40	2.76
	1.54	0.68	0.10	1.91
	2.47	0.03	0.43	1.90
Exp 3	2.93*	-0.08*	-0.16	2.62
	2.30*	0.25*	0.44	2.24
	2.04*	0.32*	-0.06	2.42

* Values are the same as in 2A Exp. 3 (first and second column) as both experiments (2A Expt. 3 and 2B Expt. 3) were performed at the same time, using these same controls.

2C

Recipient	*Δbcp-1 Δbcp-2*	*+bcpI-1*	Tn 2-2	*ΔhisD*	Tn 2-7	*ΔcspD*
Exp 1	1.61	-0.23	-0.26	1.57	-0.63	1.66
	1.67	-0.14	-0.16	2.07	-0.49	1.65
	2.34	-0.21	-0.22	1.62	-0.62	1.78
Exp 2	2.19	0.01	-0.68	2.01	-0.44	1.85
	2.16	0.07	-1.02	1.58	-0.30	1.97
	2.06	0.25	-0.57	1.89	-0.29	1.73
Exp 3	2.08**	0.16**	0.33	2.64	0.29	1.88
	2.48**	0.04**	0.54	2.58	-0.03	2.00
	2.67**	0.29**	0.16	2.49	0.00	2.00

** Values are the same as in 2D Exp. 3 (first and second column) as both experiments (2C Expt. 3 and 2D Expt. 3) were performed at the same time, using these same controls.

2D

Recipient	*Δbcp-1 Δbcp-2*	*+bcpI-1*	Tn 1-2	Δ00967
Exp 1	1.61	-0.23	-0.25	1.35
	1.67	-0.14	-0.33	1.25
	2.34	-0.21	-0.16	1.48
Exp 2	2.19	0.01	-0.40	1.11
	2.16	0.07	-0.24	1.45
	2.06	0.25	-0.25	1.21
Exp 3	2.08**	0.16**	-0.39	2.42
	2.48**	0.04**	-0.38	2.41
	2.67**	0.29**	0.41	2.44

** Values are the same as in 2C Exp. 3 (first and second column) as both experiments (2C Expt. 3 and 2D Expt. 3) were performed at the same time, using these same controls.

3A

Recipient	*Δbcp-1 Δbcp-2*	*+bcpI-2*	Δ00967	Δ00967 +00967
Exp 1	2.84 †	-0.25 †	2.32 †	3.07
	2.92 †	-0.23 †	2.47 †	2.83
	2.92 †	-0.27 †	2.54 †	2.6
Exp 2	3.06 †	0.51 †	2.76 †	1.91
	1.54 †	0.68 †	1.91 †	2.03
	2.47 †	0.03 †	1.90 †	2.05
Exp 3	2.60	-0.59	1.44	2.20
	2.41	-0.50	1.76	1.96
	2.87		1.61	1.88
Exp 4	3.41	0.36	2.60	3.34
	3.37	0.27	2.10	4.07
	3.25	0.36		3.72
Exp 5	2.59	-0.03	2.34	2.27
	2.50	0.10	2.20	1.80
	2.75	0.11	2.07	2.18
Exp 6	2.64	0.13	2.03	2.71
	2.93	-0.16	1.80	2.70
		0.00	1.87	2.87

† Values re-plotted from 2B Expt 1 and Expt 2

3B

Recipient	*Δbcp-1 Δbcp-2*	*+bcpI-1*	Δ00967	Δ00967 +00967
Exp 1	1.61 ‡	-0.23 ‡	1.35 ‡	2.02
	1.67 ‡	-0.14 ‡	1.25 ‡	1.37
	2.34 ‡	-0.21 ‡	1.48 ‡	1.34
Exp 2	2.19 ‡	0.01 ‡	1.11 ‡	2.70
	2.16 ‡	0.07 ‡	1.45 ‡	2.35
	2.06 ‡	0.25 ‡	1.21 ‡	2.64
Exp 3	3.23	0.12	1.83	1.51
	2.74	-0.46	1.71	1.48
	2.88	-0.28	1.56	1.89
Exp 4	1.60	-0.10	1.44	2.85
	2.04	-0.15	2.05	2.92
		-0.42	1.41	
Exp 5	1.75	-0.08	1.25	3.03

	1.67	0.11	1.28	2.95
	1.54	0.03	1.32	3.21
**Exp 6**	2.00	-0.02	1.15	2.10
	1.94	0.21	1.37	1.58
	2.20	-0.02	0.99	2.55

‡ Values re-plotted from 2D Expt 1 and Ept 2

3C

**Recipient**	***Δbcp-1 Δbcp-2***	***+bcpI-2***	**Tn1-2**	**Tn1-2 +00967**	**Tn1-2 +00966**	**Tn1-2 +00967-66**
**Exp 1**	3.30		-0.80	0.42	0.40	-0.19
	3.20		0.00	0.59	0.92	0.29
	3.35		-0.35	-1.01	0.81	0.09
**Exp 2**	2.96	0.05	0.03	-0.25	-1.03	0.28
	2.87	0.56	0.24	0.40	-0.96	0.03
	3.06	0.20	0.18	0.24	-0.73	0.28
**Exp 3**	1.62	-0.57	-0.11	0.58	0.44	0.26
	2.37	0.07	-0.81	0.57	-0.16	0.38
	2.55	-0.03		0.50	-0.25	0.49
**Exp 4**	2.67	-0.03	0.22	0.73	-0.27	1.21
	2.61	0.64	-0.42	0.97	-0.78	1.35
	2.54	-0.58		0.64	0.35	1.22

3D

**Recipient**	***Δbcp-1 Δbcp-2***	***+bcpI-1***	**Tn1-2**	**Tn1-2 +00967**	**Tn1-2 +00966**	**Tn1-2 +00967-66**
**Exp 1**	2.83	-0.31	-0.70	1.56	-0.46	1.84
	2.86	-0.61	-0.83	1.80	-0.20	1.90
	2.68	-0.42	-0.90	1.82	-1.41	2.22
**Exp 2**	2.82	-0.20	-0.52	0.62	0.08	0.76
	2.50	0.04	-0.61	0.39	-0.07	0.92
	1.91		-0.56	0.52	0.20	0.73
**Exp 3**	2.65	0.19	0.07	-0.21	-0.08	0.32
	2.62	0.35	-0.20	-0.15	-0.19	0.55
	2.20	-0.23	-0.29	0.12	-0.30	-0.43
**Exp 4**	3.25	-0.28	0.03	0.74	0.47	1.49
	2.94	-0.15	-0.22	1.02	0.22	1.43
	2.97	-0.28	-0.21	1.08	0.20	1.66
**Exp 5**	4.18	0.28	-0.18	0.78	0.04	0.94
	4.14	0.15	0.22	0.70	-1.08	0.58
	3.82	-0.16	0.02	0.96		0.49

4D

**Recipient**	**Δ*bcp*-1 Δ*bcp*-2**	***+bcpI*-2**	**01005-**	**01006-**
**Exp 1**	2.64	0.62	-0.08	0.17
	2.99	0.21	0.04	0.44
	2.51	0.06	0.05	0.51
**Exp 2**	2.23	-0.46	0.68	-0.17
	2.58	-0.49	0.25	-0.16
	2.25	-0.27	0.42	-0.27
**Exp 3**	2.26	0.33	0.78	0.23
	2.45	-0.29	0.63	0.55
	2.17	0.20	0.61	0.46

4E

Recipient	Δ*bcp*-1 Δ*bcp*-2	+*bcpI*-1	01005-	01006-
Exp 1	2.25	-0.21	-0.47	0.29
	2.22	-0.19	-0.25	0.07
	2.17	-0.55	-0.33	0.04
Exp 2	2.27	0.26	0.54	0.54
	2.41	0.06	0.75	0.45
	1.86	0.01	0.48	0.36
Exp 3	2.16	0.04	0.13	0.44
	2.01	-0.02	-0.27	0.22
	2.32	-0.47	0.07	0.43

4F

Recipient	Δ*bcp-1* Δ*bcp-2*	+*bcpI-1*+*bcpI-2*	01005-	01006-	Δ00967	Δ00967 01006-
Exp 1	3.87	0.17	0.51	0.57	4.46	-0.23
	4.66	0.03	0.63	0.91	4.76	0.04
	4.62	0.09	0.61	1.23		-0.45
Exp 2	3.41	0.09	0.28	0.35	4.23	0.25
	3.74	-0.15	0.47	0.6	4.32	-0.16
	4.76		0.46	0.6	4.43	
Exp 3	3.72	-0.08	0.20	0.42	3.66	0.93
	3.15	-0.08	0.41	0.57	3.02	0.75
	3.46	0.08	0.48	0.34	2.89	0.42

5C

Donor	Δ*bcp-1*	Δ*bcp-1*	Δ*bcp-1*	Δ*bcp-2*	Δ*bcp-2*	Δ*bcp-2*
Recipient	Δ*bcp-1* Δ*bcp-2*	+immunity	Δ*wabO*	Δ*bcp-1* Δ*bcp-2*	+immunity	Δ*wabO*
Exp 1	3.39	0.09	0.32	3.01	0.03	0.22
	3.39	0.24	0.48	3.02	-0.35	0.33
	3.30	0.14	0.38	2.91	-0.03	0.05
Exp 2	3.45	-0.05	0.24	2.30	-0.17	0.17
	3.41	-0.01	0.12	2.63	-0.15	0.26
	3.55	-0.19	0.28	2.63	-0.04	0.24
Exp 3	2.75	0.43	0.81	2.71	0.06	0.20
	2.53	0.32	0.47	2.49	-0.57	0.01
	2.37	-0.08	0.53	2.50	-0.06	-0.09

S1

	PS12-*lacZ*	Pneg-*lacZ*	Δ*bcp*-1 Δ*bcp*-2 PcepI-*lacZ*	Δ*bcp-1* Δ*bcp-2* Δ*cepR* PcepI-*lacZ*	Δ*bcp-1* Δ*bcp-2* Δ*cepI* PcepI-*lacZ*	Δ*bcp-1* Δ*bcp-2* Δ*cepI* +AHL PcepI-*lacZ*
Exp 1	18985	0	18939	0	6838	25640
	17586	0	16900	10	6643	23040
	16586	0	19720	0	6270	21095
Exp 2	10930	0	17813	0	5725	16916
	9920	0	16425	29	6020	17742
	12264	0	15010	0	5138	19519

S2A

Recipient	Δ*bcp*-1 Δ*bcp*-2	+*bcpI* -2	Tn 2-8	Δ*cepR*	04624-
Exp 1	2.53	0.13	1.72	1.83	1.81
	2.76	0.01	1.45	1.53	1.88
	2.43	0.38	1.73	1.87	2.1
Exp 2	2.53	-0.03	2.45	0.82	2.58

	2.09	-0.18	2.55	1.11	2.7
	2.25	-0.16	2.13	1.30	2.85
**Exp 3**	2.42	0.08	1.90	2.03	3.03
	2.76	-0.35	1.89	1.90	3.18
	1.82	-0.17	1.83	1.92	2.88

S2B

**Recipient**	**Δ*bcp*-1 Δ*bcp*-2**	***+bcpI*-2**	**Δ*cepR***	**Δ*cepR +cepR***
**Exp 1**	3.37	0.30	3.15	4.21
	3.63	0.14	2.83	3.57
	3.03		2.87	3.17
**Exp 2**	3.36	0.50	2.84	3.28
	3.93	0.82	2.90	3.75
	3.82	0.96	3.00	3.57
**Exp 3**	2.40	0.59	2.09	2.83
	2.44	0.28	1.62	2.41
	2.21	0.24	1.86	2.68

S2C

**Recipient**	**Δ*bcp*-1 Δ*bcp*-2**	***+bcpI*-2**	**Δ*cepI***	**Δ*cepI +cepI***
**Exp 1**	1.51	-0.20	1.02	2.64
	1.21	-0.22	1.22	2.88
	1.29	-0.28	1.37	2.90
**Exp 2**	1.20	0.19	0.81	1.39
	1.32	0.27	0.89	1.42
	1.40	0.09	1.10	1.26
**Exp 3**	1.64	-0.42	1.30	1.10
	1.63	0.05	1.36	1.50
	1.67	-0.26	1.13	1.58

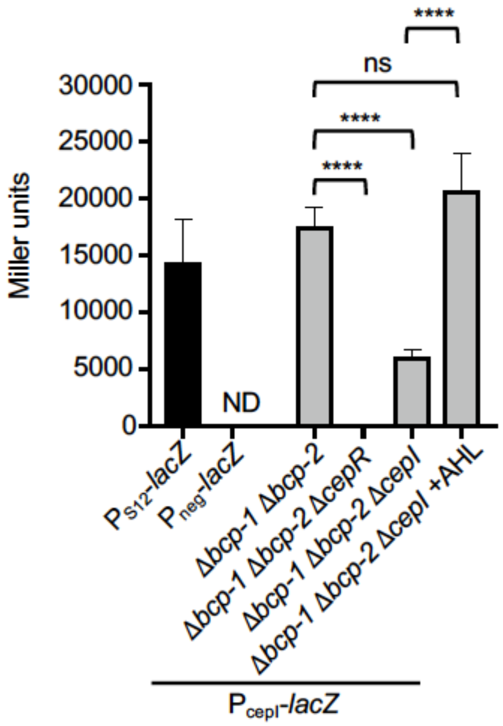

Fig. S1. Beta-galactosidase activity of a P_{cepI}-*lacZ* reporter in Δ*cepR* and Δ*cepI* mutant recipient cells. Beta-galactosidase activity (Miller units) is shown for the indicated reporter strain cultures: wild-type AU0158 carrying control reporters P_{S12}-*lacZ* (constitutive) or P_{neg}-*lacZ* (promoterless) (black bars), or P_{cepI}-*lacZ* reporter carried in Δ*bcp-1* Δ*bcp-2*, Δ*bcp-1* Δ*bcp-2* Δ*cepR,* or Δ*bcp-1* Δ*bcp-2* Δ*cepI* mutants (gray bars). Where indicated, purified C8-HSL was added to Δ*bcp-1* Δ*bcp-2* Δ*cepI* mutant cultures (+AHL). All reporters were carried in single copy at an *att*Tn7 site. Bars show the mean and SD of two independent experiments, each in triplicate (*n*=6). ND, not detected; ns, not significant; ****, $p<0.0001$.

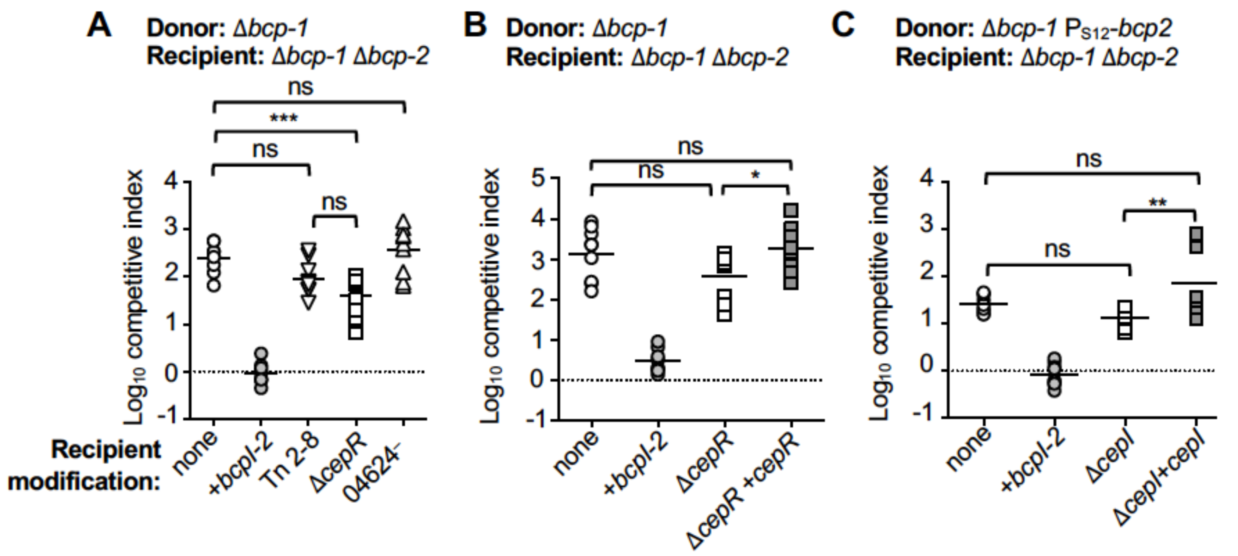

Fig. S2. Role of recipient cell *cepR and cepI* during CDI system 2-mediated interbacterial competition. (A and B) Interbacterial competition assays between Δ*bcp-1* donor bacteria and the indicated recipients: Δ*bcp-1* Δ*bcp-2* (parent; open circles), Δ*bcp-1* Δ*bcp-2* complemented with cognate *bcpI-2* (gray circles), *cepR*::miniTn5 transposon mutant (Tn 2-8; open inverse triangles), Δ*bcp-1* Δ*bcp-2* Δ*cepR (*Δ*cepR;* open squares), Δ*bcp-1* Δ*bcp-2* containing disruption mutation in BDAG_04624 (04624¯, open triangles), and Δ*bcp-1* Δ*bcp-2* Δ*cepR* complemented with P$_{S12}$-*cepR* at an *att*Tn7 site (Δ*cepR* + *cepR*; gray squares). **(C)** Interbacterial competition assays between Δ*bcp-1* mutant overexpressing *bcpAIOB-2* (P$_{S12}$-*bcp-2*) donor bacteria competed at a 1:10 (donor:recipient) ratio against the indicated recipients: Δ*bcp-1* Δ*bcp-2* (parent; open circles), Δ*bcp-1* Δ*bcp-2* complemented with cognate *bcpI-2* (gray circles), Δ*bcp1*Δ*bcp-2* Δ*cepI (*Δ*cepI;* open squares), and Δ*bcp-1* Δ*bcp-2* Δ*cepI* complemented with P$_{S12}$-*cepI*at an *att*Tn7 site (Δ*cepI* + *cepI*; light gray squares). Symbols represent log$_{10}$ competitive index values (ratio of donor to recipient) from three independent experiments and bars show the mean (*n*=8-9). Dashed line shows log$_{10}$ competitive index = 0. ns, not significant; *, *p*<0.05; **, *p*<0.01; and ***, *p*<0.001.

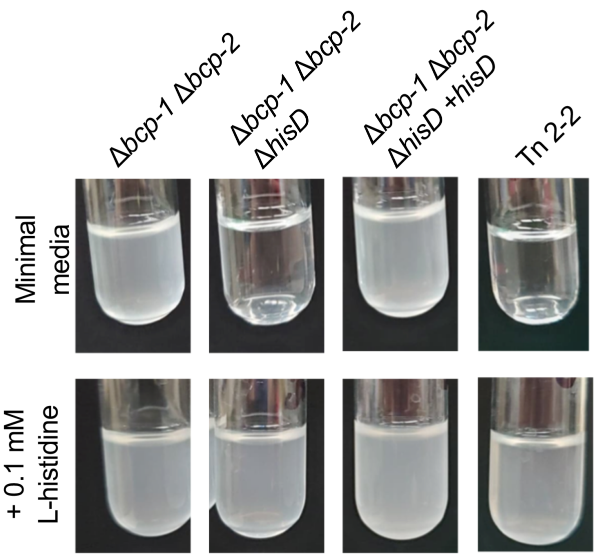

Fig. S3. Growth of *B. dolosa hisD* mutants in minimal medium. Growth of (left to right) Δ*bcp-1* Δ*bcp-2* mutant (parent), Δ*bcp-1* Δ*bcp-2* Δ*hisD* mutant, Δ*bcp-1* Δ*bcp-2* Δ*hisD* complemented with *hisD* at an *att*Tn7 site, and *hisD*:::miniTn5 (Tn 2-2) in M63 minimal medium (top) or medium supplemented with 0.1 mM L-histidine (bottom). Cultures were imaged after ~24 h aerated growth at 37°C.

Supplemental References

1. Perault AI, Cotter PA. 2018. Three distinct contact-dependent growth inhibition systems mediate interbacterial competition by the cystic fibrosis pathogen *Burkholderia dolosa*. J Bacteriol JB.00428-18.

2. López CM, Rholl DA, Trunck LS, Schweizer HP. 2009. Versatile dual-technology system for markerless allele replacement in *Burkholderia pseudomallei*. Appl Environ Microbiol 75:6496.

3. Choi K-H, Gaynor JB, White KG, Lopez C, Bosio CM, Karkhoff-Schweizer RR, Schweizer HP. 2005. A Tn7-based broad-range bacterial cloning and expression system. Nat Meth 2:443–448.

4. Choi K-H, Mima T, Casart Y, Rholl D, Kumar A, Beacham IR, Schweizer HP. 2008. Genetic tools for select-agent-compliant manipulation of *Burkholderia pseudomallei*. Appl Environ Microbiol 74:1064–1075.

5. Anderson MS, Garcia EC, Cotter PA. 2012. The *Burkholderia bcpAIOB* genes define unique classes of Two-Partner secretion and contact dependent growth inhibition systems. PLoS Genet 8:e1002877.

6. Lorenzo V de, Herrero M, Jakubzik U, Timmis KN. 1990. Mini-Tn5 transposon derivatives for insertion mutagenesis, promoter probing, and chromosomal insertion of cloned DNA in gram-negative eubacteria. J Bacteriol 172:6568–6572.

7. Norris MH, Kang Y, Wilcox B, Hoang TT. 2010. Stable, site-specific fluorescent tagging constructs optimized for *Burkholderia* species. Appl Environ Microbiol 76:7635–7640.

8. Myers-Morales T, Sim MMS, DuCote TJ, Garcia EC. 2021. *Burkholderia multivorans* requires species-specific GltJK for entry of a contact-dependent growth inhibition system protein. Mol Microbiol 116:957–973.

9. Choi K-H, DeShazer D, Schweizer HP. 2006. mini-Tn7 insertion in bacteria with multiple *glmS*-linked *att*Tn7 sites: example *Burkholderia mallei* ATCC 23344. Nat Protoc 1:162–169.
